# Glucocorticoid dose-dependent risk of type 2 diabetes in six immune-mediated inflammatory diseases: a population-based cohort analysis

**DOI:** 10.1136/bmjdrc-2020-001220

**Published:** 2020-07-27

**Authors:** Jianhua Wu, Sarah L Mackie, Mar Pujades-Rodriguez

**Affiliations:** 1 School of Dentistry, University of Leeds, Leeds, UK; 2 School of Medicine, University of Leeds, Leeds, UK

**Keywords:** adverse drug reactions, cohort, adult diabetes, glucocorticoid(s)

## Abstract

**Introduction:**

In immune-mediated inflammatory diseases, there is a lack of -estimates of glucocorticoid dose–response diabetes risk that consider changes in prescribed dose over time and disease activity.

**Research design and methods:**

Population-based longitudinal analysis of electronic health records from the UK Clinical Practice Research Datalink, linked to hospital admissions and the mortality registry (1998–2017). We included 100 722 adult patients without diabetes history, diagnosed with giant cell arteritis or polymyalgia rheumatica (n=32 593), inflammatory bowel disease (n=29 272), rheumatoid arthritis (n=28 365), vasculitis (n=6082), or systemic lupus erythematosus (n=4410). We estimated risks and HRs of type 2 diabetes associated with time-variant daily and total cumulative prednisolone-equivalent glucocorticoid dose using Cox regression methods.

**Results:**

Average patient age was 58.6 years, 65 469 (65.0%) were women and 8858 (22.6%) had a body mass index (BMI) ≥30 kg/m^2^. Overall, 8137 (8.1%) people developed type 2 diabetes after a median follow-up of 4.9 years. At 1 year, the cumulative risk of diabetes increased from 0.9% during periods of non-use to 5.0% when the daily prednisolone-equivalent dose was ≥25.0 mg. We found strong dose-dependent associations for all immune-mediated diseases, BMI levels and underlying disease duration, even after controlling for periods of active systemic inflammation. Adjusted HR for a <5.0 mg daily dose versus non-use was 1.90, 95% CI 1.44 to 2.50; range 1.70 for rheumatoid arthritis to 2.93 for inflammatory bowel disease.

**Conclusions:**

We report dose-dependent risks of type 2 diabetes associated with glucocorticoid use for six common immune-mediated inflammatory diseases. These results underline the need for regular diabetic risk assessment and testing during glucocorticoid therapy in these patients.

**Significance of this study**

**What is already known about this subject?**

- Diabetes is a known complication of glucocorticoid therapy but the magnitude of dose–response for common immune-mediated inflammatory diseases is uncertain. We report dose-dependent risks of type 2 diabetes associated with glucocorticoid use for six immune-mediated inflammatory diseases, estimated taking into account changes in prescribed medication over time.

**What are the new findings?**

- At 1 year of follow-up, the cumulative risk of diabetes was 0.9% in periods of non-use, 2.1% when the daily prednisolone-equivalent dose was <5 mg, and 5.0% when ≥25.0 mg was prescribed. We found strong dose-dependent increases in HRs for patients with any of the six diseases studied, body mass index (BMI), family history of diabetes mellitus or disease duration, even after adjusting for periods of active systemic inflammation over time.

**How might these results change the focus of research or clinical practice?**

- Patients with immune-mediated inflammatory diseases who are prescribed glucocorticoid therapy should be screened for diabetes regularly. This recommendation applies to all patients treated regardless of dose, treatment duration, family history of diabetes or BMI.

## Introduction

Glucocorticoids are often used to treat immune-mediated diseases due to their potent anti-inflammatory and immunosuppressive effects.^1 2^ Glucocorticoid therapy is the most common cause of drug-induced hyperglycemia and drug-induced diabetes mellitus.^3 4^ They might therefore have an important contributing role in the reportedly increasing global burden of diabetes.^5–8^ Glucocorticoids can unmask previously undiagnosed diabetes, trigger new (glucocorticoid-induced) diabetes, or aggravate hyperglycemia in patients with a prior diagnosis of diabetes mellitus.^9^ In a randomized controlled trial including 41 patients with early active rheumatoid arthritis, initiation of prednisolone at 60 or 30 mg/day was associated with a 24% increase in incidence of type 2 diabetes within 1 week.^10^ A recent meta-analysis of 12 studies evaluating the occurrence of diabetes in patients receiving systemic glucocorticoids reported rates of glucocorticoid-induced diabetes of 18.6%.^11^ Although not completely understood, multiple pathophysiological mechanisms are responsible for the metabolic adverse effects of glucocorticoids, including induction of peripheral insulin resistance, hepatic gluconeogenesis, and pancreatic islet-cell dysfunction.^1 12^ Blood glucose levels of non-diabetic patients sometimes normalize after discontinuing glucocorticoids but these individuals still have an increased risk of future diabetes development in these individuals.^13^

Accurate estimation of glucocorticoid-induced dose–response risk of diabetes is hindered by several factors.^1^ Patients with a variety of diseases are often treated using different glucocorticoid formulations and for differing durations. Doses are often modified depending on treatment response and disease activity. Furthermore, diagnosis of diabetes might be delayed or remain under-reported if it is based on the evaluation of fasting glucose measurements. The risk of developing diabetes depends on various factors including genetic constitution, age, comorbidity and concomitant medications. Most published studies have reported relationships with baseline medication use or dose,^4 14–16^ ignoring changing patterns in dose over time and without examining the potential role of the severity of the underlying disease treated. A cohort study conducted among patients with rheumatoid arthritis reported a 25%–30% increased risk of diabetes mellitus per each 5 mg increase of current glucocorticoids and with longer treatment duration.^17^ There is a need for well-conducted cohort studies estimating the glucocorticoid dose–response risk of diabetes mellitus among patients with different chronic inflammatory diseases treated with glucocorticoids. Such data will facilitate the evaluation of benefits of new glucocorticoid-sparing medications.

We conducted a cohort study to quantify the daily and cumulative dose-dependent risk of type 2 diabetes associated with systemic glucocorticoid use in people with six common immune-mediated inflammatory diseases. We also examined dose–response associations by type of underlying disease, body mass index (BMI) and presence or absence of family history of diabetes.

## Research design and methods

### Study setting and data

We used electronic health records from individuals registered in general practices of the Clinical Practice Research Datalink (CPRD) during the period between 1 January 1998 and 15 March 2017. CPRD gathers longitudinal data collected on demographics, health behaviors, laboratory and clinical examinations, diagnoses and complete computerized records of all drugs prescribed by general practitioners.^18^ Many studies have provided evidence of the validity of diagnostic and prescribing CPRD records for research use.^18^ Patients are largely representative of the UK population in terms of sex, age, and ethnicity.^18^ Individual records were linked to hospital admissions and the mortality registry (online supplementary text S1). Hospital records from the Hospital Episode Statistics (www.hscic.gov.uk/hes) gather diagnoses documented during hospital admission in all National Health Service hospitals in England. The Office for National Statistics (https://www.ons.gov.uk/atoz?query=mortality&size=10) provided dates and causes of death.

10.1136/bmjdrc-2020-001220.supp1 Supplementary data

### Study design and follow-up

We identified a cohort of patients aged ≥18 years, continuously registered in CPRD practices for 1 year or more, who had at least one of six prespecified immune-mediated inflammatory diseases (inflammatory bowel disease, giant cell arteritis, polymyalgia rheumatica, rheumatoid arthritis, systemic lupus erythematosus, and vasculitis). To define these diseases, we used the diagnostic codes shown in online supplementary table S1. The study follow-up started when patients first met all the inclusion criteria and ended on the date of last data collection, practice deregistration, diagnosis of diabetes or death, whichever occurred first. We excluded patients with history of diabetes (online supplementary figure S1).

### Oral glucocorticoid treatment

We identified all the prescriptions of oral, intramuscular and intra-articular glucocorticoids that were issued to the patients between 1 year prior to study start and the end dates. We obtained the daily dose from the recorded product name, which included information on product strength, directions given and quantity prescribed. We estimated the duration of each prescription by dividing the amount of tablets prescribed by the daily dose. We converted the daily dose into milligrams of prednisolone-equivalent dose to account for differences in anti-inflammatory potency for different glucocorticoid types (online supplementary table S2). We then defined several time-variant glucocorticoid exposure variables: ever use from 1 year prior to follow-up start (binary); current daily use (eg, whether the individual received or not glucocorticoids at a given time point; binary); current daily dose per 5 mg/day, with zero value when medication was not prescribed (analyzed as continuous and categorical variables (non-use, >0.0–4.9 mg, 5.0–14.9 mg, 15.0–24.9 mg, ≥25.0 mg/day)); cumulative dose since 1 year before follow-up start per 1000 mg (sum of the total dosages prescribed divided by 1000; continuous and categorical variables (non-use, >0–959 mg, 960–3054 mg, 3055–7299 mg, and ≥7300 mg; as previously defined^17 19 20^)).

### Diabetes definition

The primary outcome was the incidence of type 2 diabetes, which was defined by the date of the first recorded diagnosis (Read^21^ and International Classification of Diseases version 10 codes listed in online supplementary table S3), a recording of glycated hemoglobin (HbA1c) ≥7.0% (53 mmol/mol), or a fasting glucose result ≥7.0 mmol/L.

### Confounding factors

A priori confounders assessed at baseline were: sex, age, type of immune-mediated inflammatory disease (eg, vasculitis) and hypertension (diagnosed hypertension, ≥3 high systolic or diastolic blood pressure measurements within 1 year prior to follow-up start, or ≥2 blood pressure-lowering medication prescriptions) in online supplementary file 1. A priori time-variant confounders during follow-up were prescribed medication: inhaled, nasal, topical and rectal glucocorticoids; and disease-modifying antirheumatic drugs (DMARD) and non-steroidal anti-inflammatory drugs (NSAID) as markers of disease activity. Covariate definitions are shown in online supplementary text S1.

### Statistical analysis

We imputed missing glucocorticoid daily dose during tapering periods using multiple imputation with chained equations with generation of five data sets (online supplementary text S1). Imputation models included patient demographics (sex, age, ethnicity and index of multiple deprivation^22^), underlying immune-mediated inflammatory disease, time between follow-up start and prescription, type of glucocorticoid (eg, prednisolone), and prescribed inhaled, nasal, topical and rectal glucocorticoids.

We described baseline patient characteristics using summary descriptive statistics and used Kaplan-Meier methods to estimate cumulative probabilities of incident diabetes. We calculated rates of incidence with 95% CIs dividing the number of individuals with incident diabetes by the total number of person-years of follow-up.

We studied the effect of time-variant glucocorticoid exposure on diabetes using Cox proportional hazards models adjusted for the a priori confounders. We included the practice identifier as a random intercept to account for clustering effect, and no interaction terms. We assessed the proportional hazards assumption using Schoenfeld residual tests. We combined estimates and 95% CIs from each imputed data set using Rubin’s rules. We considered significance as p<0.05 and used two-sided tests. We used Stata V.15 (StataCorp, College Station, USA) for data management and R V.3.3.1 (http://cran.r-project.org/) for the analysis.

Secondary analyses included estimations by sex, BMI group (18.5–24.9, 25.0–29.9, ≥30.0 kg/m^2^), diabetes family history status, type of immune-mediated inflammatory disease, and according to duration of the underlying inflammatory disease at follow-up start (newly diagnosed/incident, within 2 years and over 2 years since diagnosis).

In sensitivity analyses, we further adjusted the estimates for periods of active systemic inflammation, which was defined based on prescribed glucocorticoid dose (increase in prednisolone-equivalent dose by >5 or 10 mg that sustained for over 3 weeks) and, except for patients who only had vasculitis or systemic lupus erythematosus, also on erythrocyte sedimentation rate and C-reactive protein values (≥30 mm/hour and ≥10 mg/L, respectively) (online supplementary text S1). We included this variable as a time-variant covariate to take into account changes in systemic inflammation activity over time.

The study funders had no role in the study design, data collection, analysis or interpretation, in the writing of the paper or in the decision to submit the paper for publication.

## Results

### Patient characteristics

We included 100 722 patients from 389 general practices; 32 593 (32.4%) with polymyalgia rheumatica or giant cell arteritis, 29 272 (29.1%) with inflammatory bowel disease, 28 365 (28.2%) with rheumatoid arthritis, 4410 (4.4%) with systemic lupus erythematosus, and 6082 (6.0%) with vasculitis (table 1). Patient mean age ranged from 54.5 years (SD 17.3) for inflammatory bowel disease to 72.9 (SD 10.8) for polymyalgia rheumatica or giant cell arteritis. Sixty-five percent (n=65 469) were women, 7996 (7.9%) had family history of diabetes and 8858 (22.6%) had a BMI ≥30 kg/m^2^. At follow-up start, the median duration since immune-mediated disease diagnosis was 8.0 years (IQR=4.0–13.0) and the most common comorbidities diagnosed were hypertension (29.3%), cardiovascular disease (19.1%), and asthma (15.1%).

**Table 1:** Patient baseline characteristics by type of immune-mediated inflammatory disease

	All immune-mediated diseases	Polymyalgia rheumatica or giant cell arteritis	Inflammatory bowel disease	Rheumatoid arthritis	Systemic lupus erythematosus	Vasculitis
	n=100 722	n=32 593	n=29 272	n=28 365	n=4410	n=6082
Follow-up time in years, median (IQR)	4.9 (2.1–6.0)	4.4 (1.9–5.4)	5.1 (2.1–6.4)	5.3 (2.3–6.4)	5.3 (2.1–6.4)	4.3 (1.8–5.4)
Total	668 601	204 172	199 180	198 199	30 173	36 986
Since first recorded immune-mediated disease diagnosis	8.0 (4.0, 13.0)	6.0 (3.0, 10.0)	9.0 (4.0, 16.0)	9.0 (4.0, 15.0)	9.0 (4.0, 15.0)	6.0 (3.0, 11.0)
Sociodemographic information						
Age in years, mean (SD)	58.6 (18.7)	72.9 (10.8)	45.4 (17.3)	58.9 (15.5)	47.9 (15.6)	52.8 (19.1)
Women, n (%)	65 469 (65.0)	22 699 (69.6)	15 376 (52.5)	20 232 (71.3)	3709 (84.1)	3453 (56.8)
Ethnicity, n (%)						
White	88 040 (87.4)	29 030 (89.1)	26 019 (88.9)	24 475 (86.3)	3369 (76.4)	5147 (84.6)
Asian	2595 (2.6)	342 (1.0)	986 (3.4)	788 (2.8)	283 (6.4)	196 (3.2)
Black	1012 (1.0)	124 (0.4)	240 (0.8)	332 (1.2)	249 (5.6)	67 (1.1)
Other	1138 (1.1)	193 (0.6)	434 (1.5)	310 (1.1)	133 (3.0)	68 (1.1)
Index of multiple deprivation, n (%)						
1st (least deprived)	18 061 (17.9)	6357 (19.5)	5349 (18.3)	4483 (15.8)	725 (16.4)	1147 (18.9)
5th (most deprived)	16 513 (16.4)	4195 (12.9)	4869 (16.6)	5558 (19.6)	885 (20.1)	1006 (16.5)
Biomarkers, mean (SD)						
Body mass index (kg/m^2^)	26.5 (5.6)	26.9 (5.3)	25.5 (5.4)	27.1 (5.9)	26.2 (5.6)	27.4 (6.3)
C-reactive protein (mg/L)	25.3 (38.9)	33.6 (43.4)	19.3 (38.5)	20.2 (31.0)	7.6 (13.0)	20.7 (39.3)
Erythrocyte sedimentation rate (mm/hour)	32.1 (27.4)	40.1 (29.1)	20.3 (20.9)	28.5 (24.7)	22.3 (22.5)	22.5 (24.4)
Total cholesterol (mmol/L)	5.1 (1.2)	5.1 (1.2)	5.0 (1.1)	5.2 (1.1)	5.1 (1.2)	5.1 (1.2)
LDL cholesterol (mmol/L)	3.0 (1.0)	3.0 (1.0)	3.0 (1.0)	3.1 (1.0)	3.2 (1.0)	3.0 (1.0)
Systolic blood pressure (mm Hg)	134 (19.4)	140 (18.4)	127 (17.9)	134 (19.1)	127 (18.6)	133 (19.2)
Diastolic blood pressure (mm Hg)	78 (10.3)	78 (10.1)	76 (10.2)	79 (10.4)	77 (10.5)	78 (10.6)
Family history of diabetes	7996 (7.9)	1887 (5.8)	2691 (9.2)	2399 (8.5)	484 (11.0)	535 (8.8)
Smoking status, n (%)						
Non-smoker	37 324 (37.1)	14 188 (43.5)	9791 (33.4)	9674 (34.1)	1331 (30.2)	2340 (38.5)
Ex-smoker	14 473 (14.4)	6432 (19.7)	3461 (11.8)	3493 (12.3)	305 (6.9)	782 (12.9)
Current smoker	24 099 (23.9)	6577 (20.2)	6981 (23.8)	8003 (28.2)	1119 (25.4)	1419 (23.3)
Comorbidities, n (%)						
Hypertension	29 506 (29.3)	15 137 (46.4)	4046 (13.8)	7560 (26.7)	948 (21.5)	1815 (29.8)
Cardiovascular disease	19 198 (19.1)	9628 (29.5)	2795 (9.5)	4827 (17.0)	668 (15.1)	1280 (21.0)
Asthma	15 226 (15.1)	4909 (15.1)	4574 (15.6)	4104 (14.5)	569 (12.9)	1070 (17.6)
Chronic obstructive inflammatory disease	4723 (4.7)	2215 (6.8)	745 (2.5)	1465 (5.2)	83 (1.9)	215 (3.5)
Cancer	6581 (6.5)	3169 (9.7)	1180 (4.0)	1626 (5.7)	192 (4.4)	414 (6.8)
Renal disease	2887 (2.9)	1548 (4.7)	366 (1.3)	652 (2.3)	92 (2.1)	229 (3.8)
Prescribed medication in last year, n (%)						
Blood pressure-lowering medication	33 198 (33.0)	16 807 (51.6)	4789 (16.4)	8565 (30.2)	1069 (24.2)	1968 (32.4)
Statins	13 371 (13.3)	7247 (22.2)	1771 (6.1)	3098 (10.9)	430 (9.8)	825 (13.6)
Oral, intramuscular or intra-articular glucocorticoids	17 240 (17.1)	6737 (20.7)	4318 (14.8)	4767 (16.8)	767 (17.4)	651 (10.7)
Inhaled or nasal glucocorticoids	10 270 (10.7)	3875 (12.6)	2421 (8.6)	2973 (10.9)	306 (7.2)	694 (12.0)
Rectal glucocorticoids	6159 (6.4)	691 (2.2)	4726 (16.7)	543 (2.0)	72 (1.7)	127 (2.2)
Topical glucocorticoids	2464 (2.6)	827 (2.7)	666 (2.4)	611 (2.2)	149 (3.5)	210 (3.6)
Non-steroidal anti-inflammatory drugs	46 680 (48.4)	18 024 (58.4)	5953 (21.1)	19 219 (70.5)	1558 (36.9)	1923 (33.1)
DMARDs ever during follow-up	19 723 (20.5)	1171 (3.8)	4131 (14.6)	12 726 (46.7)	1364 (32.3)	329 (5.7)

Percentage of patients with missing ethnicity, body mass index, C-reactive protein, erythrocyte sedimentation rate, total cholesterol, LDL cholesterol, systolic blood pressure, and smoking status was 7.9%, 61.0%, 68.8%, 57.9%, 73.6%, 83.9%, 47.7%, 36.6%, and 24.6%, respectively.

DMARD, disease-modifying antirheumatic drug; LDL, low-density lipoprotein.

In the year prior to follow-up start, 17 240 (17.1%) patients received oral, intramuscular or intra-articular glucocorticoids, 10 270 (10.7%) inhaled or nasal glucocorticoids and 46 680 (48.4%) NSAIDs. During follow-up 19 723 (20.5%; range 3.8% for polymyalgia or giant cell arteritis to 46.7% for rheumatoid arthritis) received DMARDs.

### Incidence and cumulative risk of type 2 diabetes

Diabetes mellitus was diagnosed in 8137 (8.1%) patients during 668 601 person-years of follow-up (median time per patient was 4.9, IQR 2.1–6.0 years; online supplementary table S4); 4727 events occurred during periods without glucocorticoid therapy. The incidence rate of diabetes was 12.2 per 1000 person-years (95% CI 11.9 to 12.4). This incidence rate increased from 9.2 (95% CI 9.0 to 9.5) per 1000 person-years during periods without prescribed glucocorticoids (unexposed periods) to 20.4 (95% CI 18.5 to 22.5) per 1000 person-years during periods with ≥25 mg daily dose; and from 12.6 (95% CI 12.1 to 13.1) per 1000 person-years during unexposed periods to 10.5 (95% CI 10.0 to 11.0) per 1000 person-years during periods when the cumulative dose was ≥7300 mg.

The cumulative risk of diabetes at 1 year increased from 0.9% (95% CI 0.8% to 1.0%) for periods without prescribed glucocorticoids, through 2.1% (95% CI 1.9% to 2.4%) for <5 mg, to 5.0% (95% CI 4.0% to 6.0%) for ≥25.0 mg daily dose (online supplementary table S5). We generally found stronger dose–response estimates in men than in women, especially for systemic lupus erythematosus and vasculitis (online supplementary tables S6 and S7).

### Association between glucocorticoid dose and type 2 diabetes

**Figure 1:**
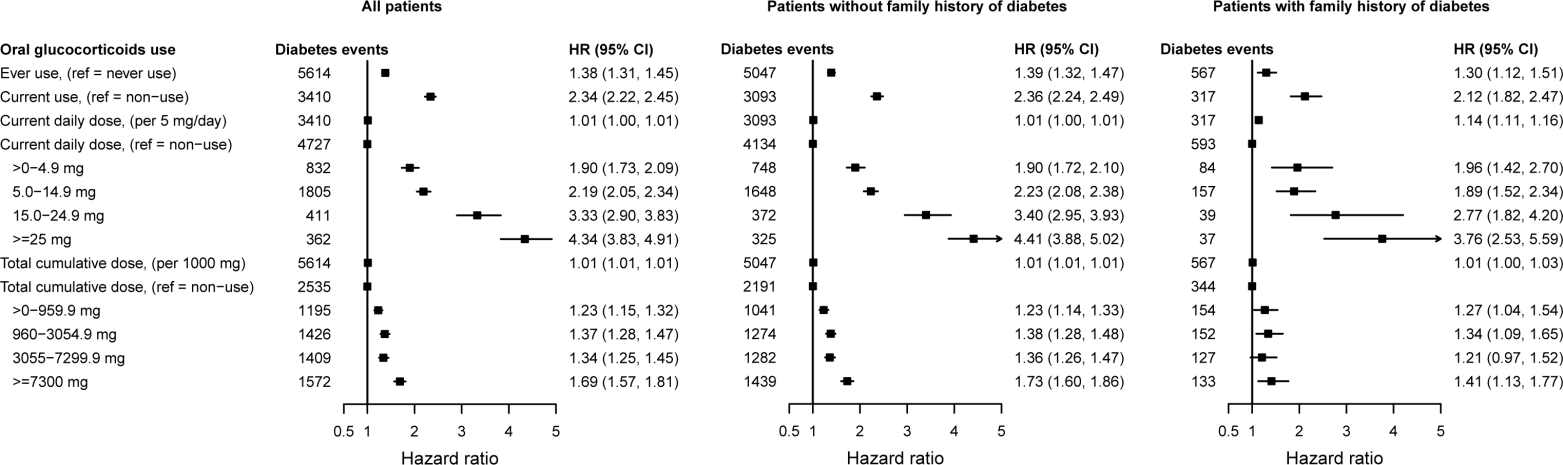
Associations between time-variant prescribed oral glucocorticoids and incident type 2 diabetes for patients with any of the six immune-mediated inflammatory diseases included. HRs from Cox proportional imputed models adjusted for baseline age, sex, hypertension, prescribed non-oral glucocorticoids and blood pressure-lowering medication, and immune-mediated inflammatory disease type; and time-variant use of disease-modifying antirheumatic drugs and non-steroidal anti-inflammatory drugs; the general practice identifier was included as a random intercept to account for clustering effect.

We observed strong dose–response effects for both daily and cumulative dose for patients with and without family history of diabetes, and for each immune-mediated inflammatory disease (figure 1 and table 2). The adjusted HR for periods with a daily dose >0–4.9 mg was 1.90 (95% CI 1.73 to 2.09); range 1.28 for vasculitis to 2.09 for inflammatory bowel disease. The overall increase in HR per 5 mg daily dose was 1.01 (95% CI 1.00 to 1.01), ranging from 1.02 (95% CI 1.01 to 1.04) for polymyalgia rheumatica or giant cell arteritis to 1.23 (95% CI 1.17 to 1.30) for systemic lupus erythematosus. This estimate was slightly higher for patients with a family history of diabetes compared with those without (1.96 (95% CI 1.42 to 2.70) vs 1.90 (95% CI 1.72 to 2.10)). Adjusted HRs were higher for daily doses <15 mg during the earlier periods following the underlying inflammatory disease diagnosis, compared with later periods (eg, 2.09 (95% CI 1.86 to 2.34) within the year of diagnosis, 1.90 (95% CI 1.44 to 2.50) within the second year of diagnosis and 1.51 (95% CI 1.24 to 1.84) after 2 years of diagnosis; online supplementary tables S8–S10). However, estimates were generally higher for daily doses ≥15 mg in longer periods after diagnosis, compared with earlier periods.

**Table 2:** Associations between time-variant prednisolone-equivalent glucocorticoid dose and incident type 2 diabetes by immune-mediated inflammatory disease

	Adjusted HRs with 95% CI				
	Polymyalgia rheumatica or giant cell arteritis	Inflammatory bowel disease	Rheumatoid arthritis	Systemic lupus erythematosus	Vasculitis
Events (n)	3236	1831	2303	276	491
Ever use (ref non-use since 1 year before follow-up start)	1.33 (1.18 to 1.51)	1.32 (1.19 to 1.45)	1.41 (1.29 to 1.54)	1.53 (1.18 to 1.99)	1.30 (1.08 to 1.57)
Current use (ref non-use)	2.32 (2.15 to 2.51)	2.86 (2.55 to 3.20)	2.01 (1.84 to 2.20)	2.71 (2.05 to 3.57)	2.20 (1.79 to 2.71)
Current daily dose per 5 mg/day	1.02 (1.01 to 1.04)	1.05 (1.04 to 1.06)	1.03 (1.02 to 1.04)	1.23 (1.17 to 1.30)	1.20 (1.15 to 1.26)
Current daily dose in mg (ref non-use)	1.00	1.00	1.00	1.00	1.00
>0–4.9	2.00 (1.79 to 2.23)	2.09 (1.53 to 2.88)	1.66 (1.37 to 2.02)	1.72 (0.93 to 3.19)	1.28 (0.52 to 3.12)
5.0–14.9	2.29 (2.07 to 2.53)	2.34 (1.93 to 2.84)	1.90 (1.71 to 2.12)	2.67 (1.94 to 3.68)	2.14 (1.57 to 2.90)
15.0–24.9	3.14 (2.64 to 3.74)	4.20 (2.90 to 6.07)	3.07 (2.28 to 4.14)	3.00 (1.05 to 8.59)	3.04 (1.89 to 4.87)
≥25	3.88 (3.20 to 4.71)	5.05 (4.09 to 6.24)	4.00 (3.08 to 5.21)	6.63 (3.55 to 12.38)	3.66 (2.21 to 6.06)
Cumulative dose per 1000 mg	1.03 (1.02 to 1.03)	1.01 (1.00 to 1.01)	1.02 (1.01 to 1.02)	1.03 (1.01 to 1.04)	1.01 (1.00 to 1.02)
Cumulative dose in mg (ref non-use)	1.00	1.00	1.00	1.00	1.00
1.0–959.9	1.12 (0.96 to 1.31)	1.25 (1.09 to 1.42)	1.17 (1.04 to 1.32)	1.20 (0.83 to 1.75)	1.24 (0.97 to 1.59)
960.0–3054.9	1.27 (1.11 to 1.46)	1.27 (1.10 to 1.46)	1.44 (1.25 to 1.65)	1.01 (0.61 to 1.69)	1.32 (0.95 to 1.85)
3055.0–7299.9	1.24 (1.08 to 1.42)	1.37 (1.17 to 1.60)	1.58 (1.38 to 1.82)	2.40 (1.64 to 3.52)	1.26 (0.92 to 1.74)
≥7300.0	1.86 (1.62 to 2.14)	1.55 (1.31 to 1.83)	1.61 (1.42 to 1.81)	1.87 (1.30 to 2.69)	1.44 (1.07 to 1.95)

HRs from Cox proportional imputed models adjusted for baseline age, sex, hypertension, prescribed non-oral glucocorticoids and blood pressure-lowering medication, and underlying inflammatory disease type; and time-variant use of disease-modifying antirheumatic drugs and non-steroidal anti-inflammatory drugs; the general practice identifier was included as a random intercept to account for clustering effect.

**Figure 2:**
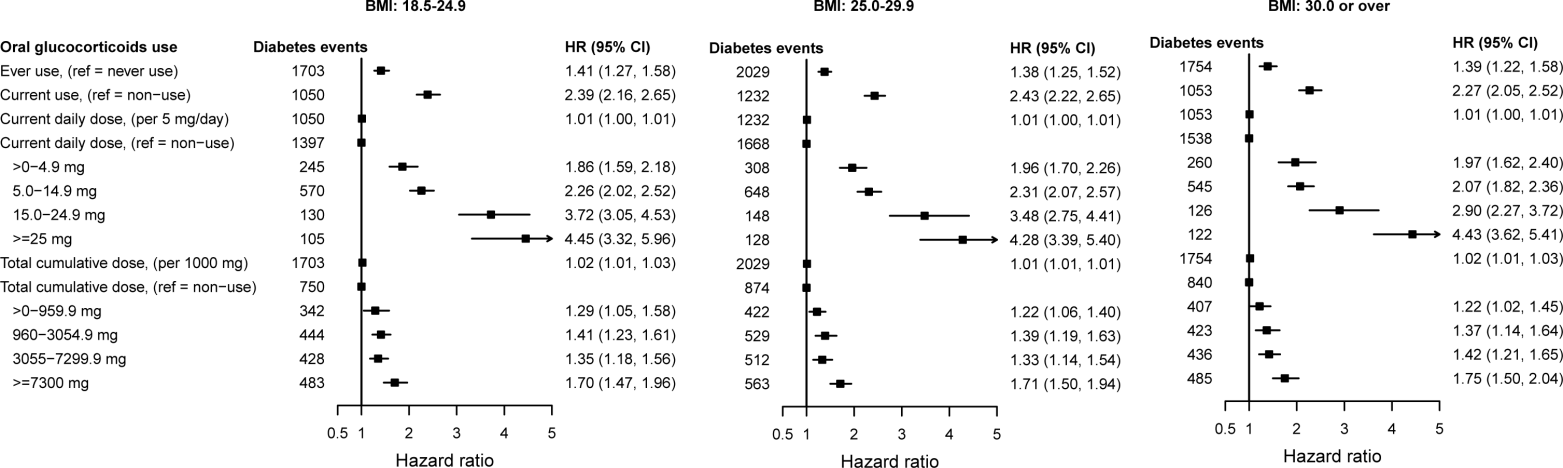
HRs with 95% CIs for the association between time-variant oral glucocorticoid dose and type 2 diabetes for patients with any of the six immune-mediated inflammatory diseases, stratified by BMI group. HRs from Cox proportional imputed models adjusted for baseline age, sex, hypertension, prescribed non-oral glucocorticoids and blood pressure-lowering medication, and immune-mediated inflammatory disease type; and time-variant use of disease-modifying antirheumatic drugs and non-steroidal anti-inflammatory drugs; the general practice identifier was included as a random intercept to account for clustering effect. BMI, body mass index.

The percentage of patients with baseline BMI 18.5–24.9 kg/m^2^, 25.0–29.9 kg/m^2^ and ≥30 kg/m^2^ who developed type 2 diabetes was 4.5%, 7.9% and 14.0%, respectively. Dose–response effects were similar regardless of BMI but, among patients with normal weight, lower estimates were observed during periods with a daily dose >0–4.9 mg and higher estimates during periods with higher daily doses, compared with patients with higher BMI (figure 2; online supplementary figures S2–S6). Further, adjustment for periods of active systemic inflammation during follow-up generally decreased the size of the dose–response estimates, but associations remained statistically significant (online supplementary tables S11 and S12).

## Conclusions

In this longitudinal study of 100 722 adults with six immune-mediated inflammatory diseases, we quantified glucocorticoid dose-dependent risks of type 2 diabetes, taking into account changes in prescribed prednisolone-equivalent dose over time. At 1 year of follow-up, the cumulative risk of diabetes was 0.9% during periods of non-use, 2.1% when the daily dose was <5 mg, and 5.0% when ≥25.0 mg of prednisolone-equivalent daily dose was prescribed. We observed strong dose-dependent increases in HRs for patients with all underlying immune-mediated diseases, irrespective of BMI, family history of diabetes mellitus or disease duration, even after adjusting for systemic inflammation activity over time.

Our findings are consistent with those from previous studies. Results from a recent meta-analysis including 12 studies evaluating the risk of hyperglycemia in patients treated with systemic glucocorticoids showed rates of glucocorticoid-induced hyperglycemia and diabetes of 32.3% and 18.6%, respectively.^11^ In a case–control study investigating the risk of diabetes requiring antidiabetic treatment, the ORs associated with <10 and >25 mg prednisolone-equivalent dose were 1.36 (95% CI 1.10 to 1.69) and 5.82 (95% CI 2.47 to 12.35), respectively.^4^ Furthermore, a longitudinal study of patients with rheumatoid arthritis found a 25%–30% increase in risk of diabetes associated with each 5 mg increase in current prednisolone-equivalent dose; only medication taken in the previous 6 months was associated with current diabetes risk.^17^ However, in that study no statistically significant difference was found for the increase in diabetes risk for a dose <5 mg, compared with non-use.

The dose-dependent increased risk of type 2 diabetes with glucocorticoid therapy highlights the need for physicians to educate patients prescribed glucocorticoids for immune-mediated inflammatory diseases on the risks of glucocorticoid-induced hyperglycemia and the action patients should take if possible symptoms of hyperglycemia occur. Physicians should also ensure regular evaluation of diabetes status, irrespective of the dose of glucocorticoid prescribed. These results also reinforce the principle that glucocorticoids should be prescribed at the lowest dose and for the shortest duration needed to control disease activity, and that appropriate glucocorticoid-sparing therapies are important to consider where such option exists.

By analyzing a cohort of all people diagnosed with six immune-mediated inflammatory diseases, irrespective of disease duration or activity level, we increased the generalizability of our findings. To prevent the introduction of time-related bias, the beginning of follow-up was unrelated to the start or use of glucocorticoid treatment and we used time-variant exposure and confounding medication variables. We improved the ascertainment of the outcome through analysis of linked electronic medical records from three data sources (primary care, hospital admissions and the death registry). We defined the outcome using diagnostic codes previously used for research^23^ and clinically reviewed by the authors, as well as HbA1c and fasting glucose measurements. Some patients might however have being missed because HbA1c testing reflects glycemia over the previous 2–3 months^12^ and because glucocorticoids can increase postprandial glucose levels without necessarily affecting fasting glucose measurements.^1^ All medication prescribed in primary care, where the majority of care occurs for patients with these six immune-mediated diseases, is contemporaneously recorded. We extracted the daily dose of oral, intra-articular and parenterally administered glucocorticoids and the duration of use from the recorded instructions on how to take the medication directions for each prescription. When these were undefined (eg, written ‘as directed’ during dose tapering) we used information on the longitudinal doses prescribed to patients to impute the dose taken. Data on drug adherence or on medication prescribed in hospitals were not available. This is likely to have led to dose underestimation during periods of outpatient or inpatient management and may have led to dose overestimation during periods of drug tapering. This misclassification is likely to have underestimated dose–response effects. Although there is no clear evidence that diabetes risk is affected by the level of systemic inflammation, we investigated its potential effect and the possibility of ascertainment bias through further adjustment for both periods of active systemic inflammation and prescribed medication (DMARDs and NSAIDs) during follow-up and conducting analyses in patient subgroups defined by the time since immune-mediated disease diagnosis. Similar dose–response patterns were obtained. We adjusted estimates of risk for basic sociodemographic factors, medication use and type of immune-mediated inflammatory disease. Dose–response estimates were similar in groups defined according to recorded family history of diabetes status or baseline BMI and after further adjusting for changing periods of active systemic inflammation.

In conclusion, we reported improved estimates of glucocorticoid-induced diabetes for people with six immune-mediated inflammatory diseases. Our results underscore the need to educate patients treated with glucocorticoids on recognition of typical symptoms and to provide adequate follow-up to ensure early identification and management of diabetes. This recommendation applies to all patients treated regardless of dose, treatment duration, family history of diabetes or BMI.
