# Supplementary material for: Glucocorticoid dose-dependent risk of type 2 diabetes in six immune-mediated inflammatory diseases: a population-based cohort analysis
Source: BMJ Open Diabetes Res Care. 2020 Jul 27;8(1):e001220. doi: 10.1136/bmjdrc-2020-001220 (PMC7389515; doi:10.1136/bmjdrc-2020-001220)
Supplement: Supplementary data [file bmjdrc-2020-001220supp001.pdf]

Glucocorticoid dose-dependent risk of type 2 diabetes in six immune-mediated inflammatory diseases: a population-based cohort analysis

Jianhua Wu, PhD[1*], Sarah L Mackie, PhD[2,3], Mar Pujades-Rodriguez, PhD[4*]

*These authors equally contributed to this work.

ONLINE-ONLY SUPPLEMENTAL MATERIAL

CONTENT

SUPPLEMENTAL TEXT

Text S1. Extended methods

SUPPLEMENTAL TABLES

Table S1. Codes used to define diagnosis of immune-mediated inflammatory diseases.

Table S2. Factors used to calculate prednisolone-equivalent dose of glucocorticoids.

Table S3. Codes used to define type 2 diabetes.

Table S4. Observation time and crude incidence rates of type 2 diabetes by sex.

Table S5. Cumulative probability of type 2 diabetes by current daily and cumulative glucocorticoid prednisolone-equivalent dose level and type of immune-mediated inflammatory disease.

Table S6. Cumulative probability of type 2 diabetes by current daily and cumulative glucocorticoid prednisolone-equivalent dose level and type of immune-mediated inflammatory disease in men.

Table S7. Cumulative probability of type 2 diabetes by current daily and cumulative glucocorticoid prednisolone-equivalent dose level and type of immune-medicated inflammatory disease in women.

Table S8. Hazard ratios with 95% confidence intervals for the association between time variant oral glucocorticoid dose and type 2 diabetes by type of immune-mediated

inflammatory diseases, restricted to patients with newly diagnosed immune-mediated inflammatory disease.

Table S9. Hazard ratios with 95% confidence intervals for the association between time variant oral glucocorticoid dose and type 2 diabetes in people with six immune-mediated inflammatory diseases, restricted to patients diagnosed with immune-mediated inflammatory disease in the previous 2 years.

Table S10. Hazard ratios with 95% confidence intervals for the association between time variant oral glucocorticoid dose and type 2 diabetes in people with six immune-mediated inflammatory diseases, restricted to patients diagnosed with immune-mediated inflammatory diseases for over 2 years.

Table S11. Hazard ratios with 95% confidence intervals for the association between time variant oral glucocorticoid dose and type 2 diabetes in people with six immune-mediated inflammatory diseases, adjusted for periods of active systemic inflammation during follow-up (defined by biomarker or 5 mg daily dose increase).

Table S12. Hazard ratios with 95% confidence intervals for the association between time variant oral glucocorticoid dose and type 2 diabetes in people with six immune-mediated inflammatory diseases, adjusted for periods of active systemic inflammation during follow-up (defined by biomarker or 10 mg daily dose increase).

SUPPLEMENTAL FIGURES

Figure S1. Study flow diagram

Figure S2. Hazard ratios with 95% confidence intervals for the association between time variant oral glucocorticoid dose and type 2 diabetes in people with polymyalgia rheumatica or giant cell arteritis, stratified by BMI group.

Figure S3. Hazard ratios with 95% confidence intervals for the association between time variant oral glucocorticoid dose and type 2 diabetes in people with inflammatory bowel disease, stratified by BMI group.

Figure S4. Hazard ratios with 95% confidence intervals for the association between time variant oral glucocorticoid dose and type 2 diabetes in people with rheumatoid arthritis, stratified by BMI group.

Figure S5. Hazard ratios with 95% confidence intervals for the association between time variant oral glucocorticoid dose and type 2 diabetes in people with systemic lupus erythematosus, stratified by BMI group.

Figure S6. Hazard ratios with 95% confidence intervals for the association between time variant oral glucocorticoid dose and type 2 diabetes in people with vasculitis, stratified by BMI group.

Wu J, *et al. BMJ Open Diab Res Care* 2020; 8:e001220. doi: 10.1136/bmjdrc-2020-001220

Text S1. EXTENDED METHODS

Sources of data

The dataset analysed included individual patient electronic health records from three data sources spanning primary and hospital care. the Clinical Practice Research Datalink (CPRD-GOLD; www.cprd.com), the Hospital Episodes Statistics (HES; http://www.hscic.gov.uk/hes) and the Office of National Statistics (ONS; http://www.ons.gov.uk/ons). Data linkage had been performed by CPRD. The CPRD dataset contains complete information on all the prescriptions issued to the patients registered in their general practices. CPRD also gathers health data recorded and coded during primary care contacts, including demographic and lifestyle information, symptoms, medical diagnoses, clinical and laboratory examinations and medical procedures. CPRD data were coded with the Read classification system (version 2), which is a hierarchical clinical classification containing over 96,00 codes. Accuracy and completeness of data collected in CPRD general practices are regularly audited. Studies have shown that, compared with the UK census, patients included in the CPRD dataset are broadly representative of the UK population, in terms of age, sex and ethnicity[1,2]. Compared to participants in the Health Survey for England (household based population survey), CPRD patients have also been shown to be comparable in terms of distribution of body mass index. Multiple validation studies of data collected in CPRD (e.g. clinical diagnoses), primarily evaluating their positive predicted value (PPV), have shown high PPV estimates; and have also reported estimates of incidence that are similar to other UK data sources[3-6]. HES provides information about medical diagnoses made during all elective and emergency hospital admission across all National Health Service hospitals in England. HES and ONS data are coded using the 10th revision of the International Classification of Diseases (ICD-10) classification system. ONS data collected prior to 2000 is coded using the 9th revision of the ICD.

Definition of covariates

To create the baseline covariates used in the analysis we used the following definitions:

- medication use: ≥1 prescriptions issued to the patient within 1 year prior to the start of follow-up in CPRD
- smoking status: the closest smoking status recorded within 1 year before the start of follow-up in CPRD. We categorised as ‘ex-smokers’ patients who had non-smoker status

at baseline but for whom 'current smoking' had been recorded at any time prior to the follow-up start.

- comorbidities: a diagnosis recorded at any time before the start of follow-up in CPRD or HES
- quantitative biomarkers (e.g. body mass index): the closest measurement recorded within 1 year before the start of follow-up in CPRD. The average of measurements was taken when more than one value was recorded on the same date.

Definition of periods of active systemic inflammation

We accounted for potential increases in risk of diabetes mellitus induced by periods of active systemic inflammation, splitting each patient's follow-up into phases of activity and inactivity. This enabled us to create a time variant binary variable with value '0' in phases of inactive systemic inflammation and value '1' during phases of activity. We defined active systemic inflammation using measurements of daily glucocorticoid dose and, for people with giant cell arteritis, polymyalgia rheumatica rheumatoid arthritis and inflammatory bowel disease, also taking into account results of C-reactive protein (CRP) and erythrocyte sedimentation rate (ESR) tests. People with systemic lupus erythematosus and vasculitis can have flares without increases of these biomarkers and, therefore, we did not consider this information for their definition of active systemic inflammation. Phases of active systemic inflammation began when: 1) the prescribed prednisolone-equivalent daily dose suddenly increased by >5 mg (or >10 mg) and lasted more than 3 weeks; 2) the CRP was ≥10 mg/L; or 3) the ESR was ≥30 mm/h. The active phase finished when the glucocorticoid daily dose decreased to <5 mg (or <10 mg) and/or the values of biomarkers decreased to normal if available (CRP <10 mg/L and/or ESR <30 mm/h).

Multiple imputation of glucocorticoid dose and covariates

Imputation of missing daily glucocorticoid dose and covariate data was performed in 2 stages.

Stage 1: Multiple imputation of glucocorticoid dose

During the follow-up, the median number of prescriptions issued per person was 18 (IQR 5-53) and the median length of time between prescriptions was 30 days (IQR 22-55). The total

Wu J, *et al. BMJ Open Diab Res Care* 2020; 8:e001220. doi: 10.1136/bmjdrc-2020-001220

number of tablets prescribed was available for all patients and prescriptions, and was used to calculate the period covered by each prescription. This was truncated at/corrected to 90 days when longer, given that general practitioners are requested not to provide medication for longer periods. The median number of prescriptions with unrecorded dose per patient (i.e. during tapering periods) was 12.1% (IQR 0.0-52.3%). After adjusting for major confounding factors (e.g. age, underlying disease, disease duration), the missing daily dose appeared to be missing at random. It was therefore imputed, by generating 5 datasets through multiple imputation with chained equations (MICE R package, version 3.3.1).

The imputation model specifications and variable missingness are shown in the table below.

**Variable**	**Variable Type**	**Missing (%)**	**Imputation method**
Patient indicator	Continuous, non-normal	0	Predictor/Auxiliary variable
Family practice indicator	Continuous, non-normal	0	Predictor/Auxiliary variable
Age	Continuous, non-normal	0	Predictor/Auxiliary variable
Sex	Binary	0	Predictor/Auxiliary variable
Ethnicity	Category	7.9	Polytomous logistic regression
Index of multiple deprivation	Continuous, non-normal	0	Predictor/Auxiliary variable
Underlying immune-mediated diseases (first recorded disease)	Category	0	Predictor/Auxiliary variable
Duration of underlying inflammatory disease (for first recorded disease)	Category	0	Predictor/Auxiliary variable
Daily oral prednisolone-equivalent glucocorticoid dose	Continuous, non-normal	35.4*	Predictive mean matching
Type of oral glucocorticoid**	Category	0	Predictor/Auxiliary variable
Time between the follow-up start and the date of glucocorticoid prescription	Continuous, non-normal	0	Predictor/Auxiliary variable
Prescribed non-oral glucocorticoids (inhaled, nasal, topical and rectal)	Binary (1 variable for each administration route)	0	Predictor/Auxiliary variable

* This indicates the total percentage of missingness in relation to the total number of prescriptions, not in relation to the total number of patients or to the total number of glucocorticoid exposure episodes/periods. The median number of prescriptions with missing dose per patient was 12.1%. ** Oral glucocorticoid drugs were: beclomethasone, betamethasone, budenoside, cortisone, deflazacort, dexamethasone, hydrocortisone, methylprednisolone, prednisolone, prednisone, and triamcinolone.

Stage 2: Multiple imputation of body mass index for secondary analyses

We performed this imputation stage separately for each of the 5 dose-imputed datasets. Information appeared to be missing at random after adjusting for major confounders (e.g. age, sex, and underlying disease conditions). Hence, multiple imputation via chained equations was performed using the MICE package in R 3.3.1, generating 5 imputed datasets for each dose-imputed dataset. The imputation model specifications and variable missingness are detailed in the table below.

**Variable**	**Variable Type**	**Missing (%)**	**Imputation method**
Age	Continuous, non-normal	0	Predictor/Auxiliary variable
Sex	Binary	0	Predictor/Auxiliary variable
Ethnicity	Category	0 (Imputed)	Predictor/Auxiliary variable
Underlying immune-mediated diseases	Category	0	Predictor/Auxiliary variable
Body mass index	Continuous, non-normal	61.0	Predictor/Auxiliary variable
Index of multiple deprivation	Continuous, non-normal	0	Predictor/Auxiliary variable
Number of hospital admissions	Continuous, non-normal	10.3	Predictive mean matching
Systolic blood pressure	Continuous, non-normal	36.6	Predictive mean matching
C-reactive protein	Continuous, non-normal	68.8	Predictive mean matching
Erythrocyte sedimentation rate	Continuous, non-normal	57.9	Predictive mean matching
Smoking status	Binary	24.6	Default imputation (missing set to no)
Family history of diabetes	Binary	0	Predictor/Auxiliary variable
Cancer	Binary	0	Predictor/Auxiliary variable
Asthma	Binary	0	Predictor/Auxiliary variable
Chronic obstructive pulmonary disease	Binary	0	Predictor/Auxiliary variable
Hypertension diagnosis	Binary	0	Predictor/Auxiliary variable
Prescribed non-oral glucocorticoids	Binary	0	Predictor/Auxiliary variable
DMARDs	Binary	0	Predictor/ Auxiliary variable
NSAIDs	Binary	0	Predictor/ Auxiliary variable

Abbreviations: DMARDs, disease-modifying anti-rheumatic drugs; NSAIDs, non-steroidal anti-inflammatory drugs.

In stages 1 and 2, we compared the density of the imputed data for each imputed dataset against the density of the observed data. Furthermore, we used the Kolmogorov-Smirnov test to compare the difference between the observations and the imputed values.

TABLES

Table S1. Codes used to define diagnosis of immune-mediated inflammatory diseases

Inflammatory chronic disease	Read codes	ICD 10 codes
**Inflammatory bowel disease**	J40..12, J4z2.00 + 17 Read codes for Crohn's disease + 8 Read codes for regional enteritis<br>J41y.00, J41y100, J41yz00, J57yA00, J57y900 + 16 Read codes for ulcerative colitis<br>4 Read codes for inflammatory bowel disease	M07.4, M07.5, M09.1, M09.2, K50.0, K50.1, K50.8, K50.9, M51
**Polymyalgia rheumatica and giant cell arteritis**	G755100, G755000, G755.00, N200.00, Nyu4100, G755z00, G755200, N20..00, N20..11	M31.5, M31.6, M35.3
**Rheumatoid arthritis**	2G27.00, 66H..13, G5y8.00, G5yA.00, H570.00, N005.00, N040N00, N040Q00, N040R00, N041.00, N042.00, N042100, N042200, N042z00, N04y000, N04y011, N04y200 + 35 Read codes for rheumatoid arthritis	I52.8, J99.0, M05, M06
**Systemic lupus erythematosus**	K01x411, M154.00, M154z00, M154700 + 15 Read codes for systemic lupus erythematosus	M32
**Vasculitis**	AD61.00, C332100, D310100, D310000, D310011, F371100, F396300, F421E00, G75..00, G750.00, G757.00, G757.12, G751000, G75z.00, G758.00, G754.11, G754.00, G752112, G752111, G76B.00, K01x300, K425200, N012.00, N012000, N012011, N012700, N012x00, N040N00	D69.0, H35.0, M05, M30, M31.3, M31.4, M31.7, M35.2

Table S2. Factors used to calculate prednisolone-equivalent dose of glucocorticoids

Glucocorticoid	10mg prednisolone-equivalent (in mg)
Betamethasone	1.5
Budenoside	1.09
Cortisone	50
Deflazacort	12
Dexamethasone	1.5
Hydrocortisone	40
Methylprednisolone	8
Prednisone	10
Triamcinolone	8

Table S3. Codes used to define type 2 diabetes

Endpoint	Read codes (version 2)	ICD 10 codes
**Diagnosis**	66A3.00, 66A4.00, 66Ao.00, 66At100, 66At111, 66o5.00, C100111, C100112, C109.13, C109000, C109011, C109012, C109100, C109111, C109112, C109200, C109211, C109212, C109300, C109312, C109400, C109411, C109412, C109500, C109511, C109512, C109600, C109611, C109612, C109700, C109711, C109712, C109900, C109911, C109912, C109A00, C109A11, C109B00, C109B11, C109B12, C109C00, C109C11, C109C12, C109D00, C109D11, C109D12, C109E00, C109E11, C109E12, C109F00, C109F11, C109F12, C109G00, C109G11, C109G12, C109H00, C109H11, C109H12, C109J00, C109J11, C109J12, C109K00, C10C.11, C10D.00, C10D.11, C10F.00, C10F.11, C10F000, C10F011, C10FJ00, C10FJ11, C10FK00, C10FK11, C10FL00, C10FL11, C10FM00, C10FM11, C10FN00, C10FN11, C10FP00, C10FP11, C10FQ00, C10FR00, C10P100, C10P111, L180600, ZC2CA00, C101y00, C10G.00, C10G000, C10H.00, C10N.00, C10N000, 7276, 9360, 13L4.11, 1IA..00, 1M8..00, 2BBF.00, 2BBK.00, 2BBL.00, 2BBI.00, 2BBo.00, 2BBP.00, 2BBQ.00, 2BBR.00, 2BBr.00, 2BBS.00, 2BBT.00, 2BBV.00, 2BBW.00, 2BBX.00, 2G51000, 2G5C.00, 2G5H.00, 2G5L.00, 2G5V.00, 2G5W.00, 42c..00, 42W3.00, 44V3.00, 66A1.00, 66A2.00, 66A5.00, 66AA.00, 66AD.00, 66AG.00, 66AH.00, 66AH200, 66AI.00, 66AJ.00, 66AJ.11, 66AJ100, 66AJz00, 66AK.00, 66AL.00, 66AM.00, 66AN.00, 66AO.00, 66AP.00, 66AQ.00, 66AQ000, 66AQ100, 66As.00, 66AT.00, 66AU.00, 66Av.00, 66AV.00, 66o1.00, 66o2.00, 679L211, 67D8.00, 67IJ100, 889A.00, 8A13.00, 8BL2.00, 8CE0.00, 8CMW700, 8CP2.00, 8H2J.00, 8H3O.00, 8HBG.00, 8HgC.00, 8Hgd.00, 8HKE.00, 8HLE.00, 8HME.00, 8I57.00, 918T.00, 93C4.00, 9b92000, 9N0n.00, 9N1i.00, 9N1o.00, 9N1Q.00, 9N1v.00, 9N2d.00, 9N4I.00, 9Nl4.00, 9NM0.00, 9NN8.00, 9OL..11, 9OLD.00, C10..00, C100.00, C100011, C100100, C100z00, C102.00, C102100, C102z00, C103100, C103y00, C103z00, C104.00, C104.11, C104100, C104y00, C104z00, C105.00, C105100, C105y00, C105z00, C106.00, C106.11, C106.12, C106.13, C106100, C106y00, C106z00, C107.00, C107.11, C107.12, C107100, C107200, C107z00, C108.00, C108000, C108100, C108200, C108300, C108400, C108500, C108600, C108700, C108800, C108900, C108A00, C108B00, C108C00, C108D00, C108E00, C108F00, C108G00, C108H00, C108J00, C108y00, C108z00, C108z00, C10A.00, C10A000, C10A100, C10A500, C10C.00, C10E.12, C10E012, C10E112, C10E212, C10E312, C10E412, C10E512, C10E612, C10E712, C10E812, C10E912, C10EA12, C10EC12, C10ED12, C10EE12, C10EF12, C10ER00, C10FS00, C10M.00, C10P.00, C10y.00, C10y100, C10yy00, C10zy00, C10zz00, C314.11, Cyu2.00, Cyu2000, Cyu2300, F171100, F345000, F35z000, F372.00, F372.11, F372.12, F372000, F372100, F372200, F381300, F381311, F3y0.00, F420.00, F420000, F420100, F420200, F420300, F420400, F420500, F420600, F420700, F420800, F420z00, F440700, F464000, G73y000, K01x100, K01x111, K08yA00, K08yA11, K27y700, Kyu0300, L180500, L180700, L180X00, M037200, M271000, M271100, M271200, N030000, N030011, N030100, Q441.00, R054200, R054300, ZC2C911, ZRbH.00, 6761, 66AX.00, L180.00, L180000, L180100, L180300, L180400, L180800, L180811, L180900, L180z00, C10B.00, C10B000, C11y000	E10-E14, G59.0, G63.2, H28.0, H36.0, M14.2, N08.3, O24
**Haemoglobin a1c***	42W1.00, 42c0.00, 42W2.00, 42c1.00, 42W3.00, 42c2.00, 42W..12, 42W5.00, 44TB.00, 44TB000, 44TB100, 44TC.00, 44TL.00, 42W..00, 42W..11, 42W4.00, 42WZ.00, 42c..00, 42c3.00, 66Ae.00, 66Ae000	

**Fasting glucose****	r10d011, r10d000, c11y300, 44T2.00, 44TK.00, 44f1.00, 44g1.00,	

Abbreviations: ICD, International Classification of Diseases (versions 9 and 10)

*CPRD entity type 275; **CPRD entity type 274

Table S4. Observation time and crude incidence rates of type 2 diabetes by sex

	All patients	Men	Women
**Incidence cases, n (%)**	8137	2979	5158
**Total person-years**	668601	231197	437404
**Time at risk (years), median [IQR]**	4.9 [2.1-6.0]	4.7 [2.0-5.8]	5.0 [2.1-6.1]
**Incidence per 1,000 person-years (95% CI)**			
**Overall**	12.2 (11.9-12.4)	12.9 (12.4-13.3)	11.8 (11.5-12.1)
**Current daily PED in mg**			
Non-use	9.2 (9.0-9.5)	10.2 (9.7-10.6)	8.8 (8.4-9.1)
>0-4.9	19.0 (17.8-20.3)	19.7 (17.6-22.2)	18.7 (17.3-20.2)
5.0-14.9	21.5 (20.6-22.5)	22.8 (21.2-24.6)	20.9 (19.8-22.0)
15.0-24.9	33.8 (30.9-37.1)	32.1 (27.3-37.6)	34.7 (31.1-38.8)
≥25	20.4 (18.5-22.5)	21.6 (18.3-25.4)	19.8 (17.4-22.3)
**Cumulative PED in mg**			
Non-use	12.6 (12.1-13.1)	14.0 (13.2-14.9)	11.8 (11.2-12.3)
1-959.9	10.6 (10.0-11.2)	12.5 (11.4-13.7)	9.7 (9.0-10.4)
960-3,054.9	14.5 (13.8-15.2)	14.1 (13.0-15.4)	14.7 (13.8-15.6)
3,055-7,299.9	13.2 (12.6-13.9)	12.8 (11.7-13.9)	13.5 (12.7-14.4)
≥7,300	10.5 (10.0-11.0)	10.6 (9.7-11.5)	10.5 (9.9-11.1)

Note: CI, confidence interval; IQR, interquartile range; PED, prednisolone-equivalent dose.

Table S5. Cumulative probability of type 2 diabetes by current daily and cumulative glucocorticoid prednisolone-equivalent dose level and type of immune-mediated inflammatory disease

	Immune-mediated inflammatory disease					
	**All disease**	**Inflammatory bowel disease**	**PMR and/or GCA**	**Rheumatoid arthritis**	**Systemic lupus erythematosus**	**Vasculitis**
Incident diabetes mellitus, n (%)	8137	1831	3236	2306	277	491
**Cumulative probability (95% CI) at 1 year**	1.5 (1.4-1.5)	0.9 (0.8-1.0)	2.3 (2.1-2.4)	1.1 (1.0-1.2)	0.7 (0.5-0.9)	1.9 (1.5-2.2)
**Current daily PED in mg**						
no-use	0.9 (0.8-0.9)	0.7 (0.6-0.8)	0.9 (0.7-1.0)	1.0 (0.8-1.1)	0.4 (0.2-0.6)	1.6 (1.3-1.9)
>0-4.9	2.1 (1.9-2.4)	2.1 (1.1-3.1)	2.4 (2.1-2.8)	1.0 (0.4-1.5)	1.1 (0.0-2.6)	0.7 (0.0-1.7)
5.0-14.9	2.6 (2.4-2.9)	2.1 (1.4-2.8)	3.1 (2.7-3.4)	1.6 (1.2-2.0)	2.5 (1.1-3.8)	3.6 (2.2-5.0)
15.0-24.9	4.8 (4.0-5.6)	3.1 (1.2-4.9)	5.7 (4.7-6.7)	2.7 (0.9-4.4)	1.2 (0.0-3.5)	2.7 (0.0-5.2)
≥25.0	5.0 (4.0-6.0)	4.1 (2.4-5.8)	6.5 (4.9-8.1)	3.0 (1.0-4.9)	0.0 (0.0-0.0)	5.7 (1.5-9.6)
**Cumulative PED in mg**						
no-use	1.1 (1.0-1.1)	0.8 (0.7-0.9)	1.8 (1.4-2.1)	1.0 (0.9-1.2)	0.5 (0.2-0.7)	1.7 (1.3-2.1)
1-959.9	1.9 (1.7-2.1)	1.2 (0.8-1.5)	3.1 (2.6-3.6)	1.2 (0.8-1.6)	0.7 (0.0-1.5)	2.8 (1.7-3.9)
960.0-3,054.9	2.5 (2.3-2.7)	1.1 (0.8-1.4)	3.2 (2.9-3.5)	1.8 (1.3-2.3)	1.6 (0.3-2.9)	3.2 (1.7-4.6)
3,055.0-7,299.9	1.2 (1.1-1.4)	0.9 (0.4-1.3)	1.4 (1.2-1.6)	0.7 (0.4-1.1)	1.4 (0.3-2.5)	0.8 (0.1-1.4)
≥7,300.0	0.9 (0.6-1.2)	0.9 (0.0-1.7)	1.3 (0.7-1.8)	0.3 (0.0-0.7)	0.6 (0.0-1.8)	1.4 (0.0-3.0)
**Cumulative probability (95% CI) at 5 years**	5.8 (5.6-5.9)	4.2 (4.0-4.5)	7.7 (7.4-8.1)	5.2 (4.9-5.4)	3.5 (2.9-4.2)	6.3 (5.6-7.0)
**Current daily PED in mg**						
no-use	4.0 (3.9-4.2)	3.5 (3.2-3.8)	4.2 (3.9-4.5)	4.3 (4.1-4.6)	2.9 (2.3-3.5)	5.2 (4.5-5.8)
>0-4.9	9.8 (9.0-10.6)	6.9 (4.7-9.1)	11.6 (10.5-12.6)	6.9 (5.4-8.5)	2.9 (0.3-5.4)	6.6 (2.6-10.4)
5.0-14.9	11.0 (10.4-11.6)	10.0 (8.3-11.8)	13.5 (12.6-14.5)	7.8 (6.9-8.8)	7.2 (4.8-9.6)	11.6 (8.7-14.3)
15.0-24.9	17.4 (15.5-19.3)	12.7 (8.2-17.0)	21.6 (18.7-24.3)	11.5 (7.4-15.3)	3.1 (0.0-7.3)	15.7 (6.9-23.6)
≥25.0	16.0 (14.0-18.0)	14.7 (11.0-18.2)	19.3 (15.7-22.8)	12.7 (9.0-16.3)	11.3 (1.2-20.3)	18.9 (9.1-27.6)
**Cumulative PED in mg**						
no-use	4.9 (4.6-5.1)	4.3 (3.9-4.6)	6.7 (5.8- 7.6)	4.9 (4.5-5.3)	3.6 (2.8-4.4)	6.2 (5.2-7.1)
1-959.9	5.4 (5.0-5.8)	4.3 (3.6-4.9)	8.4 (7.4- 9.4)	4.5 (3.9-5.2)	2.3 (1.0-3.6)	5.4 (3.9-7.0)
960.0-3,054.9	7.5 (7.0-7.9)	3.9 (3.3-4.6)	10.2 (9.5-11.0)	6.6 (5.5-7.7)	2.8 (0.9-4.7)	7.7 (4.9-10.4)
3,055.0-7,299.9	7.3 (6.8-7.7)	4.4 (3.5-5.3)	7.9 (7.3- 8.5)	7.6 (6.4-8.8)	5.9 (3.1-8.7)	8.8 (5.8-11.8)
≥7,300.0	4.8 (4.5-5.2)	3.8 (2.9-4.8)	5.3 (4.8- 5.9)	4.2 (3.5-4.9)	3.5 (1.9-5.2)	5.2 (3.4-6.9)
**Cumulative probability (95% CI) at 10 years**	11.2 (11.0-11.5)	8.7 (8.3-9.2)	13.9 (13.4-14.5)	10.9 (10.4-11.4)	8.7 (7.5-9.9)	11.9 (10.7-13.1)
**Current daily PED in mg**						

no-use	8.7 (8.4-8.9)	7.5 (7.0-7.9)	9.1 (8.6-9.7)	9.4 (8.9-9.9)	7.4 (6.2-8.7)	10.7 (9.4-12.0)
>0-4.9	17.3 (16.0-18.6)	11.9 (8.5-15.1)	19.8 (17.9-21.5)	15.2 (12.6-17.7)	7.9 (2.3-13.1)	10.2 (3.5-16.4)
5.0-14.9	19.2 (18.2-20.1)	17.8 (15.0-20.4)	23.4 (21.7-25.1)	15.5 (14.0-17.0)	12.8 (9.1-16.4)	17.2 (13.2-21.0)
15.0-24.9	29.0 (25.8-32.1)	29.3 (21.1-36.6)	31.3 (26.6-35.7)	20.8 (14.7-26.4)	22.4 (7.0-35.3)	28.2 (14.3-39.8)
≥25.0	25.0 (21.9-28.0)	25.9 (20.3-31.2)	31.1 (24.4-37.1)	18.1 (13.1-22.9)	29.3 (7.0-46.3)	18.3 (9.2-26.4)
**Cumulative PED in mg**						
no-use	10.7 (10.2-11.2)	9.4 (8.6-10.1)	13.7 (12.0-15.4)	11.1 (10.3-11.8)	8.9 (7.2-10.6)	12.9 (11.0-14.7)
1-959.9	10.1 (9.4-10.7)	8.7 (7.6-9.7)	14.2 (12.6-15.9)	9.0 (7.9-10.0)	6.6 (3.8-9.2)	10.8 (8.2-13.4)
960.0-3,054.9	12.6 (11.9-13.4)	8.0 (6.9-9.0)	17.2 (15.8-18.5)	11.5 (9.9-13.1)	5.8 (2.5-8.9)	12.2 (7.9-16.2)
3,055.0-7,299.9	12.0 (11.3-12.7)	7.8 (6.6-9.0)	12.9 (11.9-13.9)	13.2 (11.4-15.0)	16.4 (10.1-22.2)	12.5 (8.5-16.4)
≥7,300.0	10.9 (10.3-11.5)	8.1 (6.7-9.4)	12.1 (11.2-13.0)	10.7 (9.5-11.8)	8.3 (5.6-10.9)	9.5 (6.8-12.2)

Note: CI, confidence interval; GCA, giant cell arteritis; IQR, interquartile range; PED, prednisolone-equivalent dose; PMR, polymyalgia rheumatica.

Table S6. Cumulative probability of type 2 diabetes by current daily and cumulative glucocorticoid prednisolone-equivalent dose level and type of immune-mediated inflammatory disease in men

	Immune-mediated inflammatory disease					
	**All disease**	**Inflammatory bowel disease**	**PMR and/or GCA**	**Rheumatoid arthritis**	**Systemic lupus erythematosus**	**Vasculitis**
Incident diabetes mellitus, n (%)	2979	932	990	758	64	236
**Cumulative probability (95% CI) at 1 year**	1.6 (1.5-1.8)	1.0 (0.8-1.2)	2.5 (2.2-2.8)	1.4 (1.1-1.6)	1.4 (0.5-2.2)	2.1 (1.5-2.6)
**Current daily PED in mg**						
no-use	1.0 (0.9-1.2)	0.8 (0.6-0.9)	1.2 (0.9-1.5)	1.2 (0.9-1.4)	0.8 (0.1-1.5)	1.9 (1.3-2.4)
>0-4.9	2.2 (1.7-2.7)	2.6 (1.0-4.2)	2.4 (1.8-3.1)	0.8 (0.0-1.8)	3.7 (0.0-10.6)	0.7 (0.0-1.9)
5.0-14.9	2.9 (2.5-3.3)	2.5 (1.5-3.5)	3.2 (2.6-3.8)	2.3 (1.5-3.1)	5.4 (0.1-10.4)	3.0 (1.1-4.8)
15.0-24.9	4.7 (3.5-6.0)	1.2 (0.0-2.8)	6.2 (4.4-7.9)	3.7 (0.1-7.2)	-	1.5 (0.0-4.3)
≥25.0	5.8 (4.1-7.5)	4.6 (2.0-7.2)	7.1 (4.1-10.1)	3.9 (0.1-7.7)	-	8.9 (1.1-16.1)
**Cumulative PED in mg**						
no-use	1.3 (1.1-1.4)	0.9 (0.7-1.1)	2.6 (1.8-3.4)	1.3 (1.0-1.6)	0.9 (0.1-1.7)	2.1 (1.4-2.8)
1-959.9	2.3 (1.9-2.8)	1.6 (1.0-2.2)	3.6 (2.6-4.6)	1.9 (1.0-2.7)	4.5 (0.0-10.4)	2.9 (1.0-4.7)
960.0-3,054.9	2.8 (2.4-3.2)	1.2 (0.7-1.7)	3.6 (3.0-4.2)	2.6 (1.5-3.6)	6.7 (0.0-13.8)	3.2 (1.0-5.3)
3,055.0-7,299.9	1.0 (0.8-1.3)	0.6 (0.1-1.1)	1.2 (0.9-1.6)	0.5 (0.0-1.0)	-	0.6 (0.0-1.4)
≥7,300.0	0.7 (0.2-1.2)	0.4 (0.0-1.0)	1.0 (0.0-1.9)	0.6 (0.0-1.5)	-	0.8 (0.0-2.4)
**Cumulative probability (95% CI) at 5 years**	6.1 (5.8-6.4)	4.5 (4.1-4.8)	7.9 (7.3-8.5)	6.4 (5.8-6.9)	5.2 (3.4-7.0)	6.9 (5.8-8.0)
**Current daily PED in mg**						
no-use	4.4 (4.1-4.6)	3.7 (3.3-4.0)	4.2 (3.6-4.7)	5.3 (4.7-5.9)	4.8 (2.9- 6.7)	5.9 (4.7-7.0)
>0-4.9	10.9 (9.4-12.4)	6.7 (3.8-9.6)	13.0 (10.9-15.0)	8.2 (5.1-11.3)	6.8 (0.0-15.4)	10.1 (2.9-16.8)
5.0-14.9	12.1 (10.9-13.2)	11.3 (8.7-13.9)	14.1 (12.3-16.0)	9.7 (7.9-11.6)	9.4 (1.9-16.3)	11.2 (7.2-14.9)
15.0-24.9	17.4 (14.0-20.7)	11.1 (4.7-17.1)	23.2 (17.6-28.4)	13.6 (5.6-20.9)	-	12.2 (1.1-22.1)
≥25.0	17.3 (13.7-20.8)	15.6 (10.0-20.9)	20.7 (13.5-27.3)	16.5 (9.0-23.5)	-	11.2 (2.1-19.4)
**Cumulative PED in mg**						
no-use	5.6 (5.2-6.0)	4.4 (3.9-5.0)	9.7 (7.7-11.7)	6.0 (5.2-6.8)	5.3 (3.0-7.5)	7.1 (5.6-8.6)
1-959.9	6.1 (5.3-6.8)	5.0 (3.9-6.0)	8.5 (6.6-10.3)	6.0 (4.6-7.4)	6.6 (0.0-12.8)	5.3 (2.7-7.8)
960.0-3,054.9	6.9 (6.2-7.7)	4.0 (3.0-4.9)	9.1 (7.8-10.3)	8.0 (5.8-10.2)	9.7 (0.1-18.4)	6.8 (2.8-10.7)
3,055.0-7,299.9	6.9 (6.2-7.6)	4.4 (3.1-5.6)	7.4 (6.4-8.4)	8.9 (6.5-11.1)	3.1 (0.0-9.0)	8.5 (4.2-12.7)
≥7,300.0	5.3 (4.6-6.0)	4.1 (2.8-5.5)	5.8 (4.8-6.8)	5.2 (3.7-6.6)	2.5 (0.0-5.9)	6.3 (3.6-9.0)
**Cumulative probability (95% CI) at 10 years**	11.7 (11.2-12.2)	9.3 (8.6-10.0)	14.0 (13.0-15.0)	12.4 (11.4-13.4)	11.8 (8.6-14.9)	13.4 (11.5-15.3)

**Current daily PED in mg**						
no-use	9.4 (8.9-9.9)	8.1 (7.4-8.7)	9.2 (8.1-10.2)	11.1 (10.0-12.2)	11.2 (7.7-14.5)	12.4 (10.3-14.5)
>0-4.9	17.8 (15.3-20.2)	13.9 (8.5-19.0)	19.4 (15.9-22.7)	16.5 (11.2-21.4)	6.0 (0.0-13.7)	16.2 (4.1-26.7)
5.0-14.9	19.8 (18.1-21.6)	19.7 (15.7-23.6)	23.4 (20.1-26.6)	16.7 (13.9-19.5)	12.2 (3.3-20.2)	17.4 (11.7-22.7)
15.0-24.9	27.5 (21.9-32.7)	21.8 (10.4-31.7)	34.2 (25.0-42.2)	16.9 (7.0-25.9)	64.3 (0.0-91.7)	18.5 (1.6-32.5)
≥25.0	22.7 (17.9-27.2)	23.3 (15.5-30.4)	29.9 (19.0-39.3)	14.5 (7.4-21.1)	-	19.5 (2.8-33.3)
**Cumulative PED in mg**						
no-use	11.8 (11.0-12.6)	9.7 (8.6-10.7)	15.8 (12.4-19.2)	13.0 (11.5-14.5)	12.6 (8.3-16.7)	15.4 (12.3-18.3)
1-959.9	11.3 (10.1-12.5)	9.7 (8.0-11.4)	14.3 (11.0-17.5)	11.7 (9.3-14.0)	9.5 (1.6-16.7)	11.9 (7.2-16.4)
960.0-3,054.9	12.2 (11.0-13.5)	8.7 (7.1-10.4)	16.0 (13.7-18.3)	12.5 (9.3-15.7)	8.8 (0.1-16.8)	10.7 (4.5-16.4)
3,055.0-7,299.9	12.0 (10.8-13.2)	9.1 (7.2-11.1)	12.6 (10.8-14.4)	14.8 (11.3-18.2)	12.6 (0.0-29.5)	11.6 (6.0-16.9)
≥7,300.0	10.6 (9.6-11.6)	7.9 (6.1-9.7)	12.2 (10.5-13.9)	10.2 (8.2-12.3)	11.1 (2.7-18.7)	11.2 (7.0-15.2)

Note: CI, confidence interval; GCA, giant cell arteritis; IQR, interquartile range; PED, prednisolone-equivalent dose; PMR, polymyalgia rheumatica

Wu J, *et al. BMJ Open Diab Res Care* 2020; 8:e001220. doi: 10.1136/bmjdrc-2020-001220

Table S7. Cumulative probability of type 2 diabetes by current daily and cumulative glucocorticoid prednisolone-equivalent dose level and type of immune-medicated inflammatory disease in women

	Immune-mediated inflammatory disease					
	All disease	Inflammatory bowel disease	PMR and/or GCA	Rheumatoid arthritis	Systemic lupus erythematosus	Vasculitis
Incident diabetes mellitus, n (%)	5158	899	2246	1548	213	255
**Cumulative probability (95% CI) at 1 year**	1.4 (1.3-1.5)	0.8 (0.7-1.0)	2.2 (2.0-2.3)	1.0 (0.8-1.1)	0.6 (0.3-0.8)	1.7 (1.3-2.2)
**Current daily PED in mg**						
no-use	0.8 (0.7-0.8)	0.6 (0.5-0.8)	0.7 (0.5-0.9)	0.9 (0.7-1.0)	0.3 (0.1-0.5)	1.4 (1.0-1.8)
>0-4.9	2.1 (1.8-2.5)	1.6 (0.3-2.9)	2.4 (2.0-2.9)	1.0 (0.4-1.7)	0.7 (0.0-1.9)	0.8 (0.0-2.3)
5.0-14.9	2.5 (2.2-2.8)	1.7 (0.8-2.5)	3.0 (2.6-3.4)	1.2 (0.8-1.7)	2.0 (0.7-3.3)	4.2 (2.1-6.2)
15.0-24.9	4.8 (3.9-5.8)	5.0 (1.6-8.3)	5.5 (4.3-6.7)	2.2 (0.3-4.1)	1.3 (0.0-3.8)	3.8 (0.0-7.9)
≥25.0	4.6 (3.4-5.8)	3.6 (1.4-5.8)	6.2 (4.3-8.1)	2.5 (0.3-4.6)	-	3.0 (0.0-7.0)
**Cumulative PED in mg**						
no-use	0.9 (0.8-1.0)	0.7 (0.6-0.9)	1.4 (1.1-1.8)	0.9 (0.8-1.1)	0.4 (0.2-0.6)	1.5 (1.0-1.9)
1-959.9	1.7 (1.4-1.9)	0.8 (0.4-1.2)	2.9 (2.3-3.5)	0.9 (0.6-1.3)	0.3 (0.0-0.7)	2.7 (1.3-4.1)
960.0-3,054.9	2.4 (2.1-2.6)	1.0 (0.6-1.4)	3.0 (2.6-3.4)	1.5 (0.9-2.0)	0.9 (0.0-1.9)	3.2 (1.1-5.2)
3,055.0-7,299.9	1.4 (1.2-1.6)	1.2 (0.5-2.0)	1.5 (1.2-1.7)	0.8 (0.4-1.3)	1.6 (0.3-2.9)	0.9 (0.0-2.0)
≥7,300.0	1.0 (0.6-1.5)	1.8 (0.0-3.7)	1.4 (0.7-2.1)	0.2 (0.0-0.5)	0.7 (0.0-2.2)	2.2 (0.0-5.1)
**Cumulative probability (95% CI) at 5 years**	5.6 (5.4-5.8)	4.0 (3.7-4.4)	7.7 (7.3-8.1)	4.7 (4.3-5.0)	3.2 (2.6-3.9)	5.8 (4.9-6.7)
**Current daily PED in mg**						
no-use	3.8 (3.6-4.0)	3.4 (3.0-3.7)	4.1 (3.8-4.5)	4.0 (3.6-4.3)	2.5 (1.8-3.1)	4.6 (3.8-5.5)
>0-4.9	9.3 (8.4-10.3)	7.2 (3.9-10.5)	11.0 (9.8-12.2)	6.4 (4.6-8.1)	2.2 (0.0-4.6)	3.2 (0.0-7.0)
5.0-14.9	10.5 (9.8-11.2)	8.7 (6.4-11.0)	13.3 (12.2-14.4)	7.0 (5.9-8.1)	6.9 (4.3-9.5)	11.9 (7.8-15.8)
15.0-24.9	17.4 (15.0-19.8)	14.3 (7.8-20.3)	20.9 (17.5-24.0)	10.5 (5.8-14.9)	4.3 (0.0-10.0)	18.8 (5.0-30.6)
≥25.0	15.3 (12.9-17.7)	13.8 (8.9-18.4)	18.8 (14.5-22.9)	10.9 (6.7-15.0)	11.4 (1.2-20.6)	23.1 (8.2-35.5)
**Cumulative PED in mg**						
no-use	4.4 (4.2-4.7)	4.1 (3.6-4.6)	5.6 (4.6-6.5)	4.4 (4.0-4.9)	3.2 (2.3-4.0)	5.4 (4.3-6.6)
1-959.9	5.1 (4.6-5.5)	3.7 (2.9-4.5)	8.3 (7.2-9.5)	4.0 (3.3-4.7)	1.7 (0.5-3.0)	5.5 (3.5-7.5)
960.0-3,054.9	7.8 (7.2-8.4)	3.9 (3.0-4.7)	10.8 (9.8-11.8)	6.0 (4.8-7.2)	1.9 (0.2-3.5)	8.3 (4.5-11.9)
3,055.0-7,299.9	7.4 (6.9-8.0)	4.4 (3.2-5.6)	8.2 (7.4-8.9)	7.0 (5.7-8.4)	6.3 (3.2-9.3)	9.0 (4.8-13.1)
≥7,300.0	4.6 (4.2-5.0)	3.5 (2.2-4.9)	5.2 (4.6-5.8)	3.8 (3.0-4.6)	3.7 (1.8-5.5)	4.0 (1.7-6.1)
**Cumulative probability (95% CI) at 10 years**	11.0 (10.7-11.3)	8.2 (7.6-8.8)	13.9 (13.3-14.6)	10.3 (9.8-10.9)	8.1 (6.8-9.4)	10.7 (9.2-12.2)

**Current daily PED in mg**						
no-use	8.3 (7.9-8.6)	6.9 (6.3- 7.5)	9.1 (8.5-9.8)	8.7 (8.1-9.4)	6.6 (5.3-7.9)	9.5 (7.9-11.1)
>0-4.9	17.1 (15.5-18.6)	9.9 (5.8-13.7)	19.8 (17.6-21.9)	14.8 (11.8-17.7)	7.8 (1.7-13.5)	3.4 (0.0-7.3)
5.0-14.9	18.8 (17.6-20.0)	15.7 (12.0-19.3)	23.3 (21.3-25.3)	15.0 (13.2-16.8)	13.0 (8.8-16.9)	16.9 (11.3-22.2)
15.0-24.9	29.7 (25.7-33.4)	33.3 (22.3-42.7)	30.0 (24.5-35.1)	22.6 (14.9-29.5)	16.3 (2.4-28.3)	36.1 (14.3-52.3)
≥25.0 mg	26.4 (22.3-30.3)	28.0 (19.8-35.4)	31.8 (23.2-39.6)	19.6 (13.1-25.7)	30.0 (7.6-46.9)	17.6 (6.6-27.3)
**Cumulative PED in mg**						
no-use	10.1 (9.5-10.6)	9.1 (8.1-10.1)	12.8 (10.8-14.8)	10.3 (9.4-11.2)	7.8 (6.0-9.6)	10.9 (8.6-13.2)
1-959.9	9.5 (8.7-10.2)	7.8 (6.4-9.2)	14.2 (12.3-16.2)	8.0 (6.9- 9.2)	6.1 (3.2-9.0)	10.2 (7.0-13.2)
960.0-3,054.9	12.8 (11.9-13.8)	7.4 (6.0-8.7)	17.7 (16.1-19.3)	11.1 (9.2-13.0)	5.3 (1.8-8.7)	13.2 (7.3-18.6)
3,055.0-7,299.9	12.0 (11.1-12.8)	6.6 (5.1-8.1)	13.1 (11.9-14.2)	12.6 (10.4-14.7)	17.0 (10.3-23.2)	13.4 (7.4-18.9)
≥7,300.0	11.0 (10.2-11.7)	8.2 (6.3-10.1)	12.0 (11.0-13.1)	10.8 (9.4-12.2)	7.9 (5.1-10.6)	7.8 (4.4-11.1)

Note: CI, confidence interval; GCA, giant cell arteritis; IQR, interquartile range; PED, prednisolone-equivalent dose; PMR, polymyalgia rheumatica

Wu J, *et al. BMJ Open Diab Res Care* 2020; 8:e001220. doi: 10.1136/bmjdrc-2020-001220

Table S8. Hazard ratios with 95% confidence intervals for the association between time variant oral glucocorticoid dose and type 2 diabetes in people with newly diagnosed immune-mediated inflammatory disease

	Adjusted hazard ratios with 95% CI					
	All diseases*	PMR and/or GCA	Inflammatory bowel disease	Rheumatoid arthritis	Systemic lupus erythematosus	Vasculitis
**No. of events**	4404	2366	747	998	51	242
**Ever use** (ref. no used since 1 year prior to study entry)	1.33 (1.23-1.44)	1.24 (1.05-1.47)	1.28 (1.10-1.50)	1.39 (1.22-1.59)	1.98 (1.04- 3.76)	1.16 (0.88-1.53)
**Current use** (ref. non-use)	2.42 (2.25-2.59)	2.38 (2.16-2.61)	2.97 (2.50-3.54)	2.08 (1.81-2.40)	4.04 (1.79- 9.10)	2.07 (1.41-3.04)
**Current daily dose per 5 mg/day**	1.01 (1.00-1.01)	1.00 (1.00-1.01)	1.05 (1.03-1.06)	1.13 (1.10-1.15)	1.33 (1.04- 1.69)	1.21 (1.12-1.32)
**Current daily dose category in mg** (ref. no-use)	1.00	1.00	1.00	1.00	1.00	1.00
<0-4.9	2.09 (1.86-2.34)	2.13 (1.88-2.42)	2.25 (1.47-3.45)	1.77 (1.29-2.42)	-	0.87 (0.10-7.16)
5.0-14.9	2.27 (2.06-2.50)	2.31 (2.05-2.61)	2.59 (1.94-3.45)	1.88 (1.57-2.24)	4.77 (1.84-12.35)	1.93 (1.06-3.53)
15.0-24.9	3.16 (2.69-3.71)	3.04 (2.54-3.63)	3.69 (2.10-6.49)	3.41 (2.06-5.64)	6.52 (0.83-51.43)	1.38 (0.34-5.61)
≥25	4.14 (3.53-4.86)	3.82 (3.09-4.72)	4.81 (3.47-6.67)	4.16 (2.92-5.95)	-	4.53 (2.21-9.28)
**Cumulative dose per 1000 mg**	1.02 (1.02-1.03)	1.03 (1.03-1.04)	1.02 (1.01-1.04)	1.02 (1.00-1.03)	1.05 (1.00- 1.09)	0.97 (0.91-1.03)
**Cumulative dose category in mg** (ref. no-use)	1.00	1.00	1.00	1.00	1.00	1.00
<0-959.9	1.22 (1.10-1.34)	1.01 (0.83-1.23)	1.26 (1.03-1.54)	1.24 (1.04-1.47)	1.30 (0.53- 3.23)	1.25 (0.91-1.71)
960-3054.9	1.34 (1.21-1.48)	1.19 (1.00-1.43)	1.18 (0.95-1.46)	1.35 (1.10-1.66)	1.15 (0.27- 4.80)	1.37 (0.82-2.27)
3055-7299.9	1.32 (1.18-1.46)	1.20 (1.00-1.44)	1.32 (1.02-1.72)	1.70 (1.37-2.11)	6.24 (2.48-15.72)	0.82 (0.40-1.68)
≥7300	1.77 (1.58-1.98)	1.81 (1.50-2.19)	1.75 (1.30-2.34)	1.54 (1.23-1.93)	3.12 (0.90-10.83)	0.62 (0.23-1.70)

Note: CI, confidence interval; GCA, giant cell arteritis; PMR, polymyalgia rheumatica; Hazard ratios from Cox proportional imputed models adjusted for baseline age, sex, hypertension, high blood pressure, prescribed non-oral glucocorticoids and blood pressure lowering medication, and immune-mediated inflammatory disease (for overall estimate); and time variant use of disease-modifying anti-rheumatic drugs and non-steroidal anti-inflammatory drugs; the general practice identifier was included as a random intercept to account for clustering effect.

Table S9. Hazard ratios with 95% confidence intervals for the association between time variant oral glucocorticoid dose and type 2 diabetes in people with 6 immune-mediated inflammatory diseases, restricted to patients diagnosed with immune-mediated inflammatory diseases in the previous 2 years

	Adjusted hazard ratios with 95% CI					
	**All diseases***	**PMR and/or GCA**	**Inflammatory bowel disease**	**Rheumatoid arthritis**	**Systemic lupus erythematosus**	**Vasculitis**
**No. of events**	1010	358	220	316	59	57
**Ever use** (ref. no used since 1 year prior to study entry)	1.36 (1.17-1.57)	1.31 (0.88-1.93)	1.53 (1.15- 2.04)	1.41 (1.12-1.78)	1.19 (0.67- 2.10)	1.12 (0.60- 2.08)
**Current use** (ref. non-use)	2.29 (1.99-2.63)	2.18 (1.74-2.72)	3.48 (2.53- 4.78)	2.11 (1.64-2.72)	2.56 (1.34- 4.90)	1.57 (0.79- 3.12)
**Current daily dose per 5 mg/day**	1.00 (1.00-1.01)	1.00 (1.00-1.01)	1.09 (0.95- 1.25)	1.20 (1.14-1.27)	1.16 (1.01- 1.33)	1.15 (0.94- 1.41)
**Current daily dose category in mg** (ref. no-use)	1.00	1.00	1.00	1.00	1.00	1.00
>0-4.9	1.90 (1.44-2.50)	1.92 (1.34-2.75)	2.93 (1.20- 7.16)	1.70 (1.03-2.80)	2.03 (0.62- 6.66)	-
5.0-14.9	2.13 (1.75-2.58)	2.13 (1.57-2.90)	2.68 (1.61- 4.46)	1.99 (1.46-2.72)	2.60 (1.21- 5.58)	1.46 (0.68- 3.17)
15.0-24.9	3.68 (2.52-5.39)	2.89 (1.39-6.01)	6.02 (2.98-12.16)	3.45 (1.46-8.15)	-	4.53 (1.51-13.61)
≥25	4.13 (2.92-5.83)	3.96 (2.13-7.34)	5.34 (2.82-10.11)	4.05 (2.06-7.97)	4.22 (0.61-29.29)	-
**Cumulative dose per 1000 mg**	1.01 (1.00-1.01)	1.02 (1.00-1.03)	1.00 (1.00- 1.01)	1.02 (1.01-1.04)	1.03 (1.00- 1.07)	1.01 (0.97- 1.05)
**Cumulative dose category in mg** (ref. no-use)	1.00	1.00	1.00	1.00	1.00	1.00
1-959.9	1.19 (0.97-1.45)	1.15 (0.70-1.88)	1.36 (0.93- 1.99)	1.22 (0.87-1.69)	0.85 (0.36- 2.01)	0.55 (0.16- 1.86)
960-3054.9	1.32 (1.07-1.62)	1.16 (0.74-1.82)	1.46 (0.99- 2.17)	1.51 (1.04-2.20)	0.73 (0.22- 2.47)	1.37 (0.53- 3.55)
3055-7299.9	1.37 (1.12-1.67)	1.20 (0.78-1.83)	1.82 (1.19- 2.78)	1.20 (0.80-1.82)	3.03 (1.41- 6.48)	1.64 (0.67- 3.99)
≥7300	1.63 (1.34-1.98)	1.68 (1.10-2.56)	1.76 (1.09- 2.84)	1.76 (1.27-2.43)	0.96 (0.39- 2.36)	1.22 (0.52- 2.86)

Note: CI, confidence interval; GCA, giant cell arteritis; PMR, polymyalgia rheumatica; Hazard ratios from Cox proportional imputed models adjusted for baseline age, sex, hypertension, high blood pressure, prescribed non-oral glucocorticoids and blood pressure lowering medication, and immune-mediated inflammatory disease (for overall estimate); and time variant use of disease-modifying anti-rheumatic drugs and non-steroidal anti-inflammatory drugs; the general practice identifier was included as a random intercept to account for clustering effect.

Table S10. Hazard ratios with 95% confidence intervals for the association between time variant oral glucocorticoid dose and type 2 diabetes in people with 6 immune-mediated inflammatory diseases, restricted to patients diagnosed with immune-mediated inflammatory diseases for over 2 years

	Adjusted hazard ratios with 95% CI					
	**All diseases***	**PMR and/or GCA**	**Inflammatory bowel disease**	**Rheumatoid arthritis**	**Systemic lupus erythematosus**	**Vasculitis**
**No. of events**	2586	512	864	989	115	106
**Ever use** (ref. no used since 1 year prior to study entry)	1.35 (1.24-1.46)	1.34 (1.09-1.65)	1.31 (1.14-1.51)	1.44 (1.26-1.65)	1.20 (0.80- 1.81)	1.27 (0.84- 1.94)
**Current use** (ref. non-use)	2.08 (1.91-2.27)	2.04 (1.70-2.43)	2.63 (2.23-3.09)	1.94 (1.70-2.21)	1.84 (1.21- 2.79)	1.95 (1.26- 3.04)
**Current daily dose per 5 mg/day**	1.01 (1.01-1.01)	1.22 (1.17-1.28)	1.15 (1.09-1.22)	1.01 (1.00-1.01)	1.23 (1.11- 1.36)	1.16 (1.02- 1.33)
**Current daily dose category in mg** (ref. no-use)	1.00	1.00	1.00	1.00	1.00	1.00
>0-4.9	1.51 (1.24-1.84)	1.45 (1.09-1.92)	1.79 (1.16-2.76)	1.57 (1.13-2.18)	1.29 (0.46- 3.63)	1.18 (0.33- 4.16)
5.0-14.9	1.98 (1.78-2.20)	2.21 (1.79-2.73)	2.07 (1.61-2.66)	1.90 (1.60-2.25)	1.67 (1.01- 2.77)	2.08 (1.21- 3.59)
15.0-24.9	3.28 (2.58-4.17)	3.36 (2.25-5.03)	4.14 (2.52-6.80)	2.65 (1.68-4.16)	2.47 (0.57-10.72)	2.98 (0.91- 9.72)
≥25	4.28 (3.46-5.29)	3.54 (1.89-6.64)	5.13 (3.84-6.87)	3.80 (2.52-5.73)	5.51 (2.08-14.57)	1.62 (0.21-12.79)
**Cumulative dose per 1000 mg**	1.02 (1.01-1.02)	1.03 (1.01-1.04)	1.01 (1.00-1.02)	1.02 (1.01-1.02)	1.01 (0.99- 1.03)	1.01 (1.00- 1.03)
**Cumulative dose category in mg** (ref. no-use)	1.00	1.00	1.00	1.00	1.00	1.00
1-959.9	1.15 (1.02-1.30)	1.13 (0.83-1.54)	1.21 (1.00-1.48)	1.08 (0.88-1.32)	1.05 (0.58- 1.90)	1.26 (0.71- 2.24)
960-3054.9	1.30 (1.14-1.48)	1.15 (0.86-1.53)	1.32 (1.07-1.62)	1.54 (1.24-1.93)	0.65 (0.28- 1.55)	1.31 (0.60- 2.84)
3055-7299.9	1.41 (1.24-1.59)	1.28 (0.98-1.67)	1.35 (1.07-1.69)	1.63 (1.33-2.00)	1.61 (0.86- 3.01)	1.26 (0.65- 2.47)
≥7300	1.55 (1.39-1.73)	1.86 (1.43-2.41)	1.44 (1.15-1.81)	1.60 (1.35-1.89)	1.46 (0.86- 2.45)	1.28 (0.71- 2.31)

Note: CI, confidence interval; GCA, giant cell arteritis; PMR, polymyalgia rheumatica; Hazard ratios from Cox proportional imputed models adjusted for baseline age, sex, hypertension, prescribed non-oral glucocorticoids and blood pressure lowering medication, and immune-mediated inflammatory disease (for overall estimate); and time variant use of disease-modifying anti-rheumatic drugs and non-steroidal anti-inflammatory drugs; the general practice identifier was included as a random intercept to account for clustering effect.

Table S11. Hazard ratios with 95% confidence intervals for the association between time variant oral glucocorticoid dose and type 2 diabetes in people with 6 immune-mediated inflammatory diseases, adjusted for periods of active systemic inflammation during follow-up (defined by biomarker or 5 mg daily dose increase)

	Adjusted hazard ratios with 95% CI					
	**All diseases***	**PMR and/or GCA**	**Inflammatory bowel disease**	**Rheumatoid arthritis**	**Systemic lupus erythematosus**	**Vasculitis**
**No. of events**	8137	3236	1831	2303	276	491
**Ever use** (ref. no used since 1 year prior to study entry)	1.17 (1.10-1.26)	1.11 (0.96-1.29)	1.12 (0.97-1.29)	1.22 (1.11-1.35)	1.62 (0.97- 2.71)	1.45 (1.04-2.03)
**Current use** (ref. non-use)	2.25 (2.14-2.37)	2.28 (2.11-2.47)	2.77 (2.45-3.12)	1.88 (1.71-2.06)	2.79 (2.02- 3.84)	2.36 (1.87-2.98)
**Current daily dose per 5 mg/day**	1.01 (1.00-1.01)	1.00 (1.00-1.01)	1.05 (1.04-1.05)	1.01 (1.01-1.01)	1.22 (1.15- 1.29)	1.20 (1.14-1.26)
**Current daily dose category in mg** (ref. no-use)	1.00	1.00	1.00	1.00	1.00	1.00
>0-4.9	1.84 (1.68-2.02)	1.96 (1.76-2.19)	2.03 (1.47-2.80)	1.56 (1.28-1.90)	1.78 (0.95- 3.31)	1.37 (0.56-3.36)
5.0-14.9	2.11 (1.97-2.26)	2.25 (2.03-2.50)	2.27 (1.86-2.76)	1.78 (1.60-1.98)	2.76 (1.93- 3.96)	2.28 (1.64-3.17)
15.0-24.9	3.21 (2.79-3.69)	3.09 (2.59-3.68)	4.05 (2.80-5.86)	2.84 (2.10-3.83)	3.11 (1.04- 9.27)	3.24 (1.99-5.26)
≥25	4.18 (3.68-4.74)	3.82 (3.15-4.63)	4.89 (3.94-6.07)	3.70 (2.84-4.83)	6.86 (3.61-13.04)	3.91 (2.33-6.55)
**Cumulative dose per 1000 mg**	1.01 (1.01-1.01)	1.03 (1.02-1.03)	1.00 (1.00-1.01)	1.01 (1.01-1.02)	1.02 (1.01- 1.04)	1.01 (1.00-1.02)
**Cumulative dose category** (ref. no-use)	1.00	1.00	1.00	1.00	1.00	1.00
1-959.9	1.07 (0.99-1.16)	0.98 (0.83-1.16)	1.07 (0.91-1.27)	1.01 (0.89-1.15)	1.34 (0.76- 2.37)	1.40 (0.97-2.02)
960-3054.9	1.17 (1.08-1.27)	1.08 (0.92-1.27)	1.08 (0.91-1.29)	1.24 (1.07-1.43)	1.13 (0.57- 2.21)	1.49 (0.98-2.27)
3055-7299.9	1.14 (1.04-1.24)	1.04 (0.88-1.22)	1.16 (0.95-1.40)	1.37 (1.18-1.58)	2.68 (1.52- 4.75)	1.45 (0.94-2.24)
≥7300	1.44 (1.32-1.56)	1.56 (1.33-1.85)	1.31 (1.07-1.60)	1.40 (1.23-1.60)	2.12 (1.16- 3.86)	1.68 (1.09-2.59)

Note: CI, confidence interval; GCA, giant cell arteritis; PMR, polymyalgia rheumatica; Hazard ratios from Cox proportional imputed models adjusted for baseline age, sex, hypertension, prescribed non-oral glucocorticoids and blood pressure lowering medication, immune-mediated inflammatory disease (for overall estimate); and time variant use of disease-modifying anti-rheumatic drugs and non-steroidal anti-inflammatory drugs; the general practice identifier was included as a random intercept to account for clustering effect.

Wu J, *et al. BMJ Open Diab Res Care* 2020; 8:e001220. doi: 10.1136/bmjdrc-2020-001220

Table S12. Hazard ratios with 95% confidence intervals for the association between time variant oral glucocorticoid dose and type 2 diabetes in people with 6 immune-mediated inflammatory diseases, adjusted for periods of active systemic inflammation during follow-up (defined by biomarker or 10 mg daily dose increase)

	Adjusted hazard ratios with 95% CI					
	**All diseases***	**PMR and/or GCA**	**Inflammatory bowel disease**	**Rheumatoid arthritis**	**Systemic lupus erythematosus**	**Vasculitis**
**No. of events**	8137	3236	1831	2303	276	491
**Ever use** (ref. no used since 1 year prior to study entry)	1.20 (1.13-1.28)	1.14 (0.99-1.30)	1.11 (0.98-1.27)	1.26 (1.15-1.39)	1.47 (0.98- 2.20)	1.26 (0.92-1.71)
**Current use** (ref. non-use)	2.24 (2.13-2.36)	2.26 (2.09-2.44)	2.74 (2.43-3.08)	1.90 (1.73-2.08)	2.74 (2.00- 3.76)	2.27 (1.78-2.90)
**Current daily dose per 5 mg/day**	1.01 (1.00-1.01)	1.00 (1.00-1.01)	1.05 (1.04-1.05)	1.01 (1.01-1.01)	1.22 (1.15- 1.29)	1.20 (1.14-1.26)
**Current daily dose category in mg** (ref. no-use)	1.00	1.00	1.00	1.00	1.00	1.00
>0-4.9	1.83 (1.67-2.01)	1.95 (1.75-2.17)	2.01 (1.46-2.77)	1.58 (1.30-1.91)	1.75 (0.94- 3.26)	1.31 (0.53-3.24)
5.0-14.9	2.11 (1.97-2.25)	2.23 (2.01-2.48)	2.25 (1.85-2.73)	1.80 (1.61-2.01)	2.73 (1.91- 3.89)	2.20 (1.59-3.04)
15.0-24.9	3.18 (2.76-3.68)	3.05 (2.56-3.65)	4.01 (2.77-5.81)	2.85 (2.11-3.85)	3.07 (1.03- 9.14)	3.12 (1.92-5.07)
≥25	4.14 (3.66-4.69)	3.77 (3.11-4.56)	4.84 (3.89-6.02)	3.71 (2.85-4.82)	6.79 (3.58-12.90)	3.79 (2.19-6.54)
**Cumulative dose per 1000 mg**	1.01 (1.01-1.01)	1.03 (1.02-1.03)	1.00 (1.00-1.01)	1.01 (1.01-1.02)	1.02 (1.01- 1.04)	1.01 (0.99-1.02)
**Cumulative dose category in mg** (ref. no-use)	1.00	1.00	1.00	1.00	1.00	1.00
1-959.9	1.10 (1.02-1.18)	1.01 (0.86-1.18)	1.08 (0.93-1.26)	1.05 (0.92-1.19)	1.21 (0.75- 1.95)	1.22 (0.87-1.70)
960-3054.9	1.20 (1.11-1.30)	1.12 (0.96-1.29)	1.08 (0.92-1.27)	1.29 (1.11-1.49)	1.02 (0.56- 1.85)	1.30 (0.86-1.95)
3055-7299.9	1.16 (1.07-1.26)	1.06 (0.91-1.23)	1.15 (0.95-1.38)	1.41 (1.22-1.63)	2.42 (1.50- 3.92)	1.24 (0.81-1.89)
≥7300	1.46 (1.34-1.58)	1.58 (1.35-1.85)	1.29 (1.06-1.56)	1.44 (1.27-1.64)	1.89 (1.12- 3.18)	1.41 (0.91-2.19)

Note: CI, confidence interval; GCA, giant cell arteritis; PMR, polymyalgia rheumatica; Hazard ratios from Cox proportional imputed models adjusted for baseline age, sex, hypertension, prescribed non-oral glucocorticoids and blood pressure lowering medication, and immune-mediated inflammatory disease (for overall estimate); and time variant use of disease-modifying anti-rheumatic drugs and non-steroidal anti-inflammatory drugs; the general practice identifier was included as a random intercept to account for clustering effect.

Wu J, *et al. BMJ Open Diab Res Care* 2020; 8:e001220. doi: 10.1136/bmjdrc-2020-001220

FIGURES

Figure S1. Study flow diagram

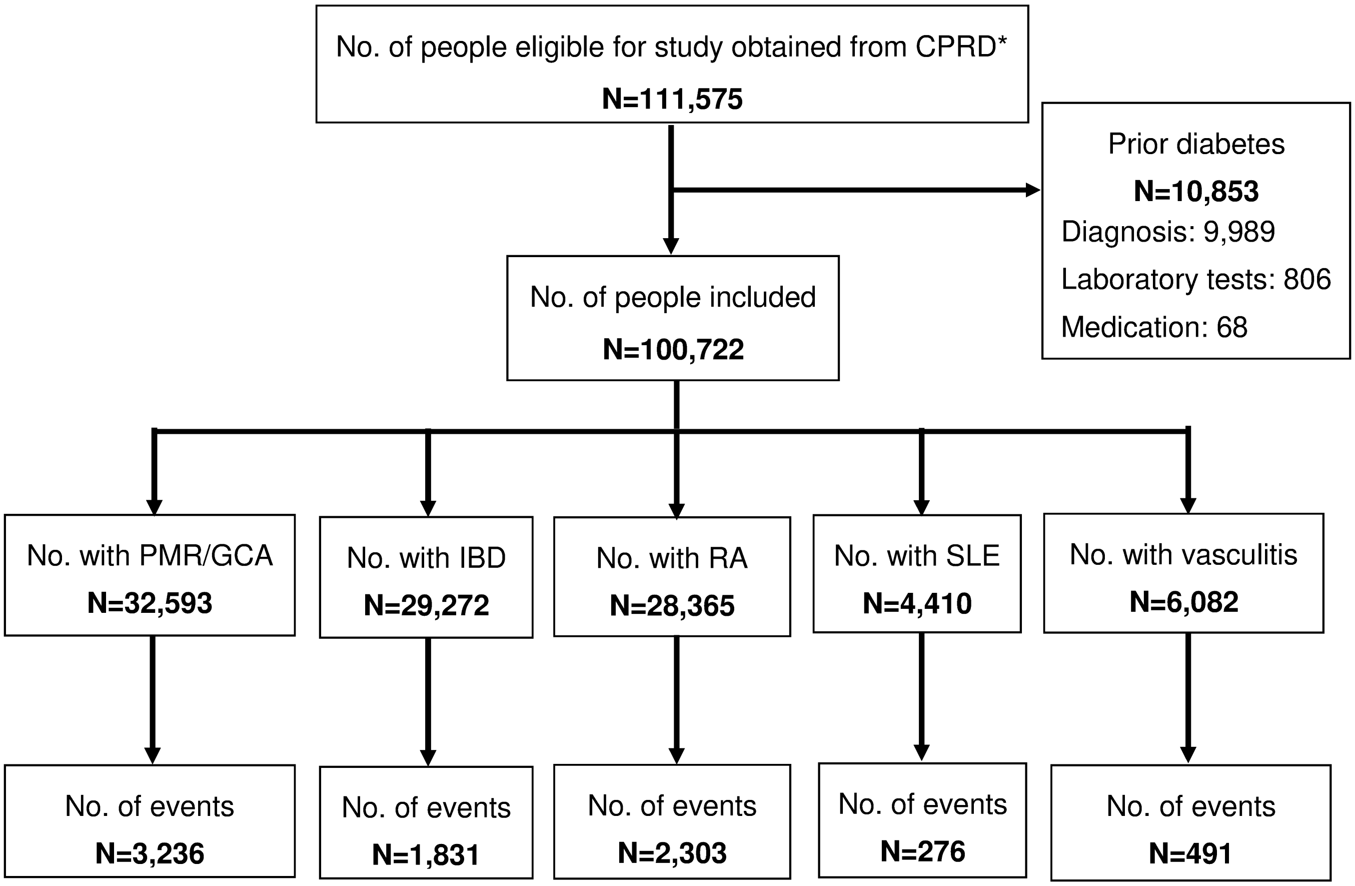

* All eligibility study inclusion criteria except for a history of diabetes mellitus

Note: CPRD, the Clinical Practice Research Datalink; IBD, inflammatory bowel disease; PMR/GCA, polymyalgia rheumatica and/or giant cell arteritis; RA, rheumatoid arthritis; SLE, systemic lupus erythematosus

Wu J, *et al. BMJ Open Diab Res Care* 2020; 8:e001220. doi: 10.1136/bmjdrc-2020-001220

Figure S2. Hazard ratios with 95% confidence intervals for the association between time variant oral glucocorticoid dose and type 2 diabetes in people with polymyalgia rheumatica or giant cell arteritis, stratified by BMI group

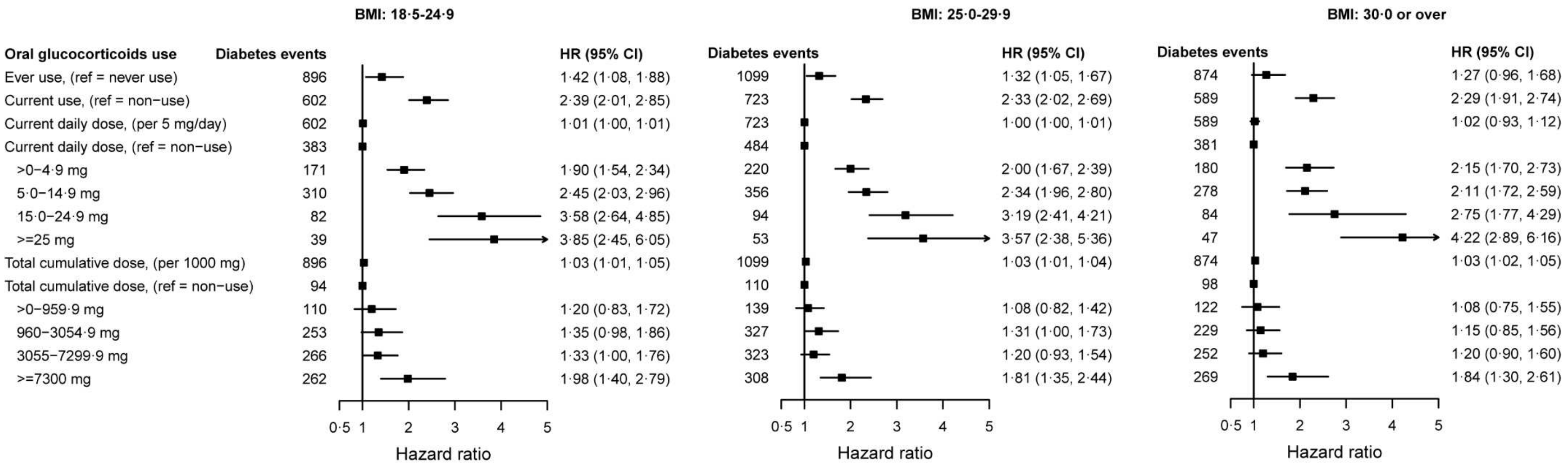

Note: BMI, body mass index; CI, confidence interval; GCA, giant cell arteritis; PMR, polymyalgia rheumatica; Hazard ratios from Cox proportional imputed models adjusted for baseline age, sex, hypertension, prescribed non-oral glucocorticoids and blood pressure lowering medication, underlying inflammatory disease duration; and time variant use of disease-modifying anti-rheumatic drugs and non-steroidal anti-inflammatory drugs; the general practice identifier was included as a random intercept to account for clustering effect.

Wu J, *et al. BMJ Open Diab Res Care* 2020; 8:e001220. doi: 10.1136/bmjdrc-2020-001220

Figure S3. Hazard ratios with 95% confidence intervals for the association between time variant oral glucocorticoid dose and type 2 diabetes in people with inflammatory bowel disease, stratified by BMI group

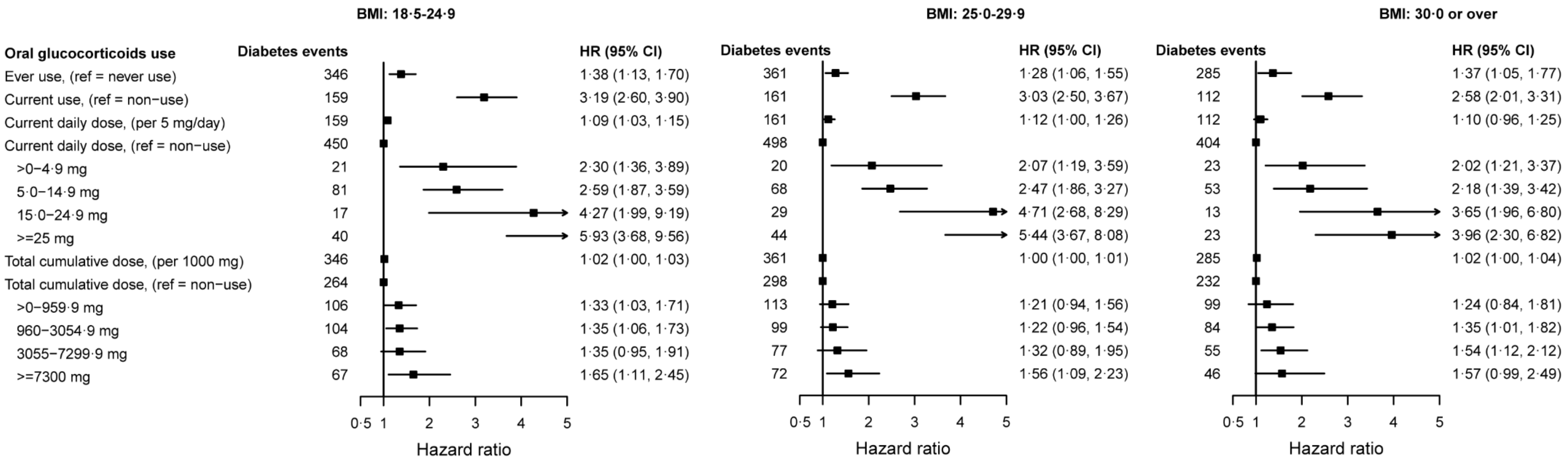

Note: BMI, body mass index; CI, confidence interval; GCA, giant cell arteritis; PMR, polymyalgia rheumatica; Hazard ratios from Cox proportional imputed models adjusted for baseline age, sex, hypertension, prescribed non-oral glucocorticoids and blood pressure lowering medication, underlying inflammatory disease duration; and time variant use of disease-modifying anti-rheumatic drugs and non-steroidal anti-inflammatory drugs; the general practice identifier was included as a random intercept to account for clustering effect.

Wu J, *et al. BMJ Open Diab Res Care* 2020; 8:e001220. doi: 10.1136/bmjdrc-2020-001220

Figure S4. Hazard ratios with 95% confidence intervals for the association between time variant oral glucocorticoid dose and type 2 diabetes in people with <u>rheumatoid arthritis</u>, stratified by BMI group

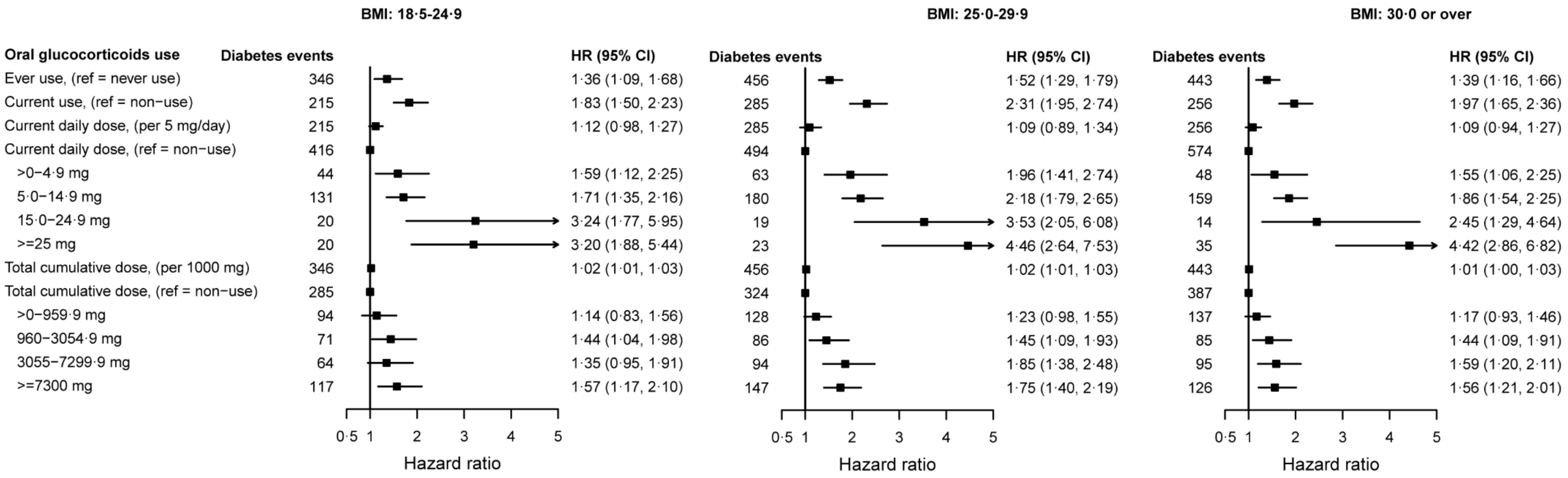

Note: BMI, body mass index; CI, confidence interval; GCA, giant cell arteritis; PMR, polymyalgia rheumatica; Hazard ratios from Cox proportional imputed models adjusted for baseline age, sex, hypertension, prescribed non-oral glucocorticoids and blood pressure lowering medication, underlying inflammatory disease duration; and time variant use of disease-modifying anti-rheumatic drugs and non-steroidal anti-inflammatory drugs; the general practice identifier was included as a random intercept to account for clustering effect.

Figure S5. Hazard ratios with 95% confidence intervals for the association between time variant oral glucocorticoid dose and type 2 diabetes in people with systemic lupus erythematosus, stratified by BMI group

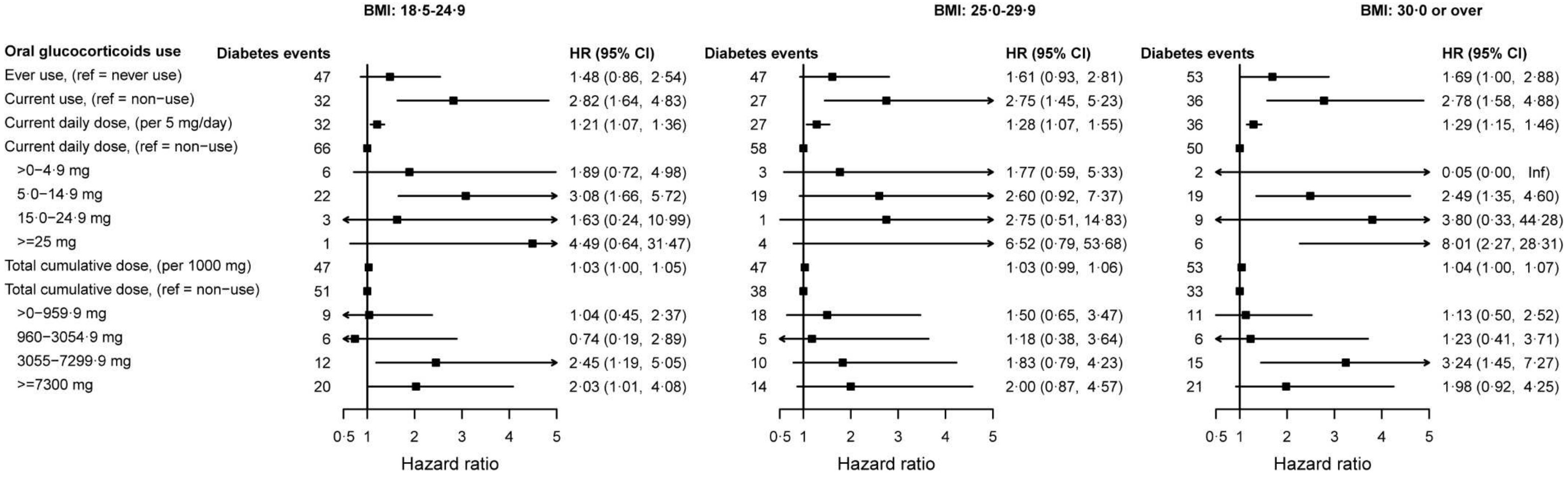

Note: BMI, body mass index; CI, confidence interval; GCA, giant cell arteritis; PMR, polymyalgia rheumatica; Hazard ratios from Cox proportional imputed models adjusted for baseline age, sex, hypertension, prescribed non-oral glucocorticoids and blood pressure lowering medication, underlying inflammatory disease duration; and time variant use of disease-modifying anti-rheumatic drugs and non-steroidal anti-inflammatory drugs; the general practice identifier was included as a random intercept to account for clustering effect.

Wu J, *et al. BMJ Open Diab Res Care* 2020; 8:e001220. doi: 10.1136/bmjdrc-2020-001220

Figure S6. Hazard ratios with 95% confidence intervals for the association between time variant oral glucocorticoid dose and type 2 diabetes in people with <u>vasculitis</u>, stratified by BMI group

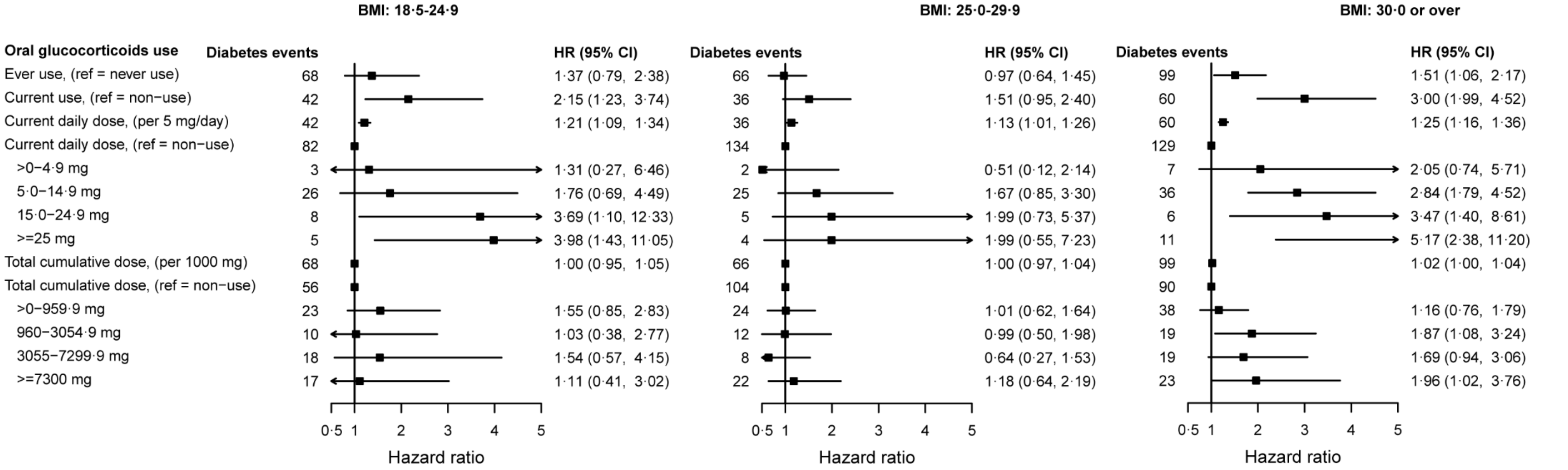

Note: BMI, body mass index; CI, confidence interval; GCA, giant cell arteritis; PMR, polymyalgia rheumatica; Hazard ratios from Cox proportional imputed models adjusted for baseline age, sex, hypertension, prescribed non-oral glucocorticoids and blood pressure lowering medication, underlying inflammatory disease duration; and time variant use of disease-modifying anti-rheumatic drugs and non-steroidal anti-inflammatory drugs; the general practice identifier was included as a random intercept to account for clustering effect.

Wu J, *et al. BMJ Open Diab Res Care* 2020; 8:e001220. doi: 10.1136/bmjdrc-2020-001220
